# Supplementary figures and images for: The redox-responsive transcriptional regulator Rex represses fermentative metabolism and is required for Listeria monocytogenes pathogenesis
Source: PLoS Pathog. 2021 Aug 16;17(8):e1009379. doi: 10.1371/journal.ppat.1009379 (PMC8389512; doi:10.1371/journal.ppat.1009379)

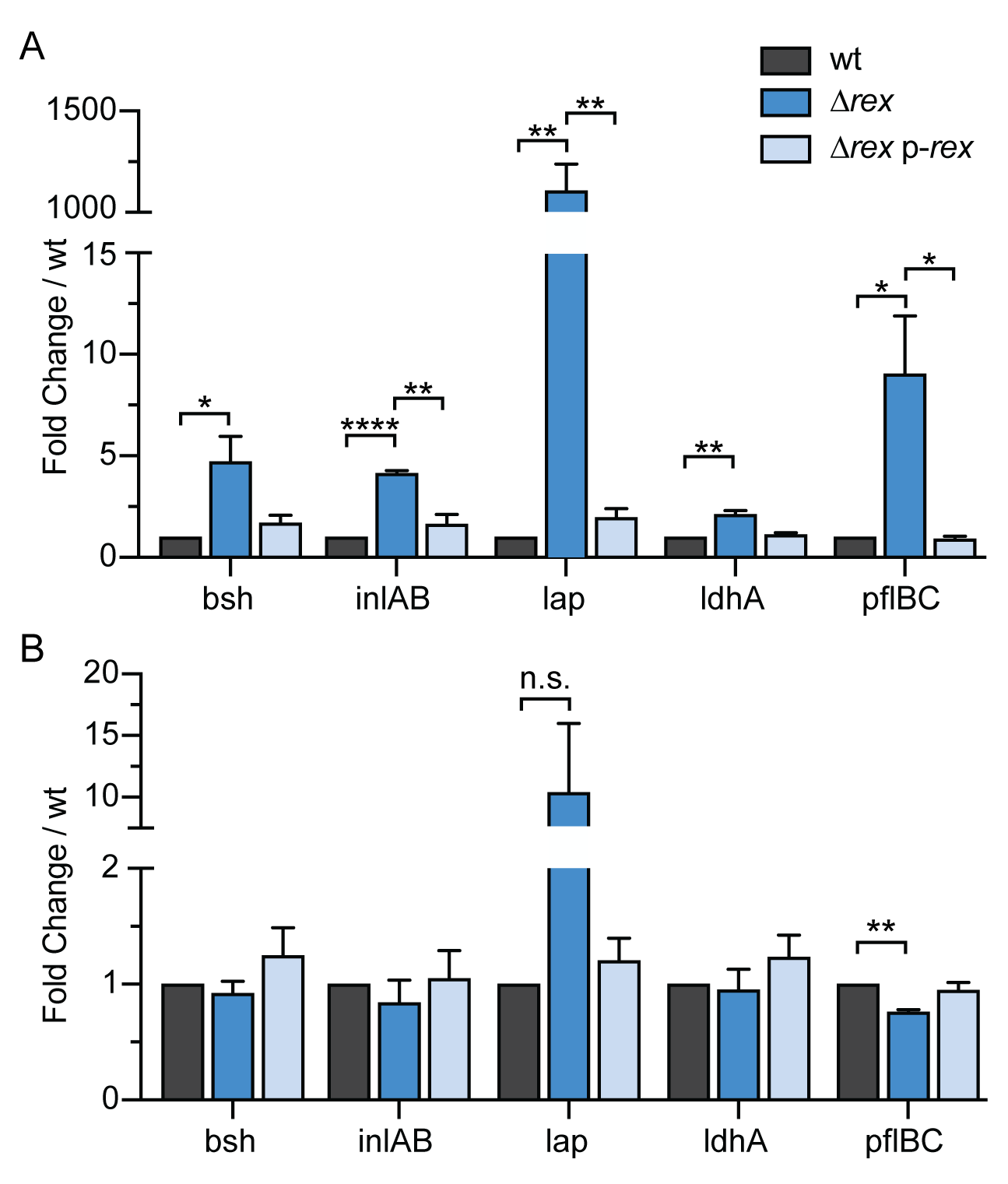

Supplement: S1 Fig — Gene expression measured by quantitative RT-PCR following 7 hours of aerobic (A) and anaerobic (B) growth in the wt, Δrex, and Δrex p-rex strains. Data are graphed as the fold change over wt (wt = 1). In both panels, data are the means and SEMs of three independent experiments. Student’s unpaired t test was used to compare fold changes between the Δrex and wt strains and between the Δrex and Δrex p-rex strains (n.s., p > 0.05; *, p < 0.05; **, p < 0.01; ****, p < 0.0001). Data were not statistically significant between wt and Δrex p-rex. (TIF) [file ppat.1009379.s001.tif]

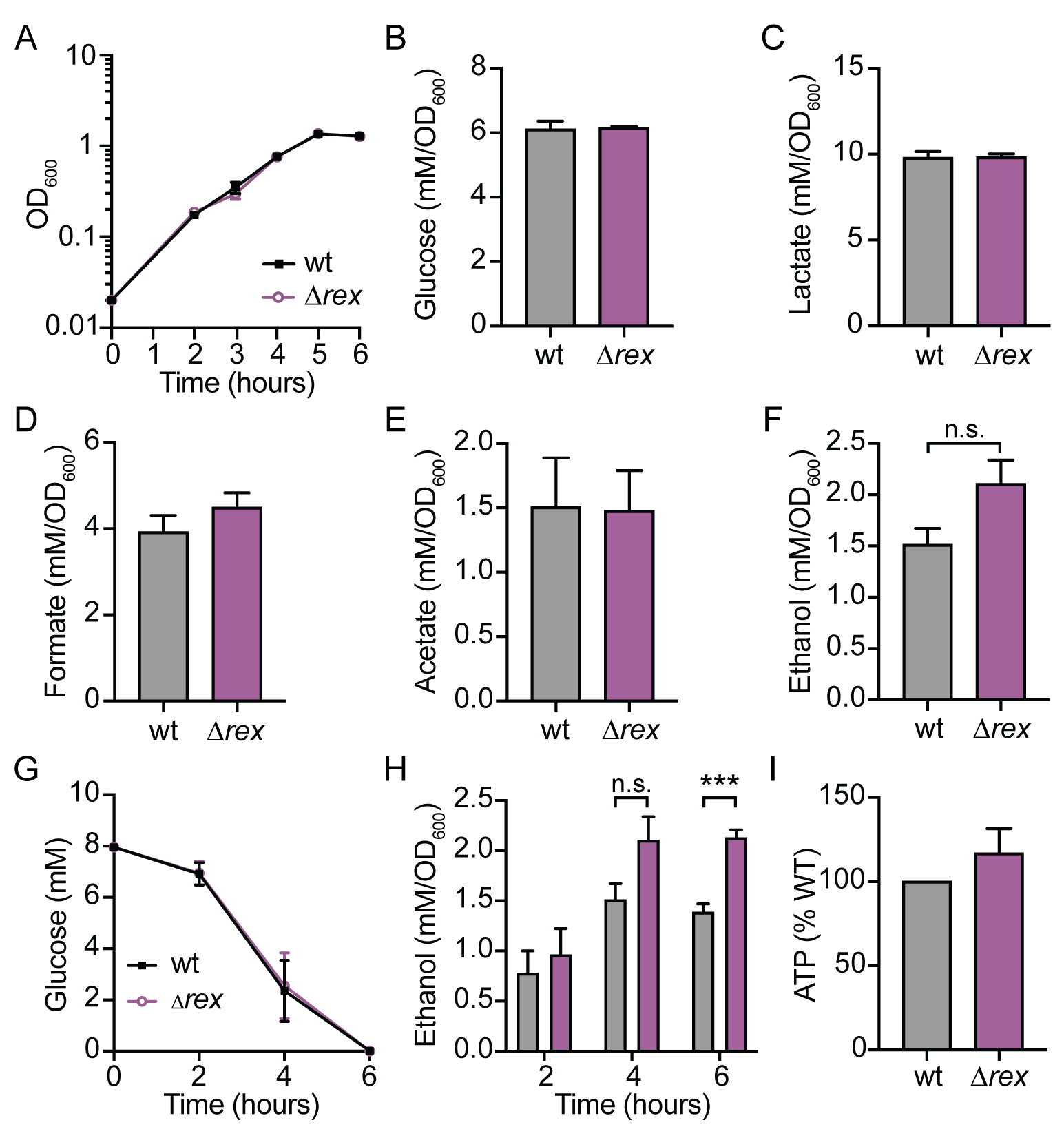

Supplement: S2 Fig — A. Anaerobic growth of wt and Δrex strains, measured by OD600. B-F. Supernatants were sampled at 4 hours during anaerobic growth. Concentrations of glucose (B), lactate (C), formate (D), acetate (E), and ethanol (F) were determined and normalized to OD600. G. Concentration of glucose was measured in the supernatant over time. H. Concentration of ethanol in the supernatant over time, normalized to the OD600 I. Relative intracellular ATP concentration was measured at 4 hours. In panels A-H, data are the means and SEMs of three independent experiments. Data in panel I is the mean and SEM of 2 independent experiments. A heteroscedastic Student’s unpaired t test was used to compare results from wt and Δrex (n.s., p > 0.05; ****, p < 0.0001). (TIF) [file ppat.1009379.s002.tif]

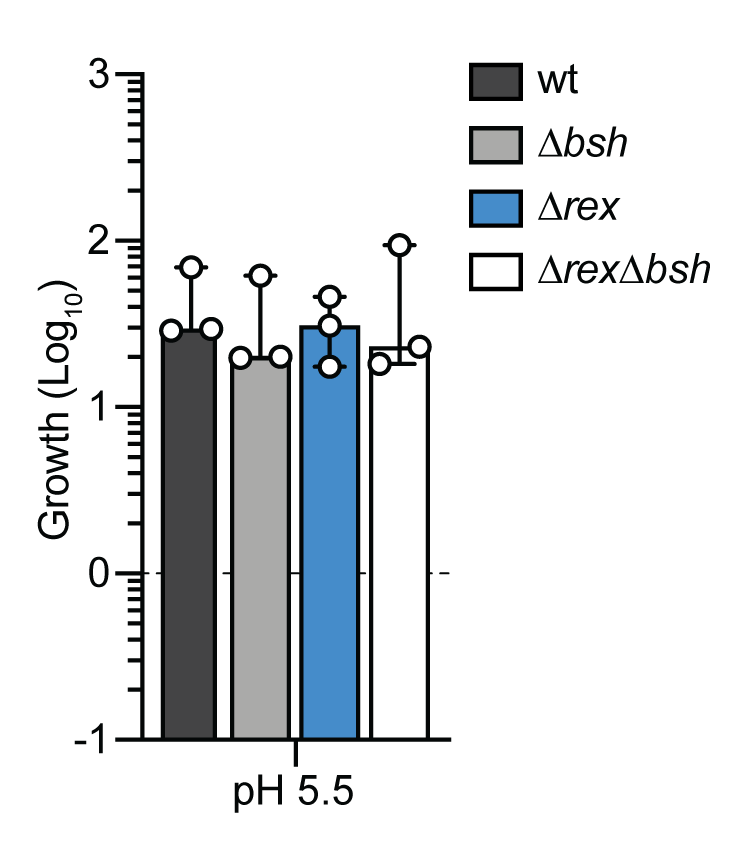

Supplement: S3 Fig — Growth of wt (black), Δbsh (grey), Δrex (blue), and ΔrexΔbsh (white) normalized to the initial inoculum (dashed line = 1). Strains were evaluated 24 hours post-inoculation in acidified BHI grown aerobically. Data are the means and range of three independent experiments. Strains were not significantly different (heteroscedastic Student’s t test; p > 0.05). (TIF) [file ppat.1009379.s003.tif]

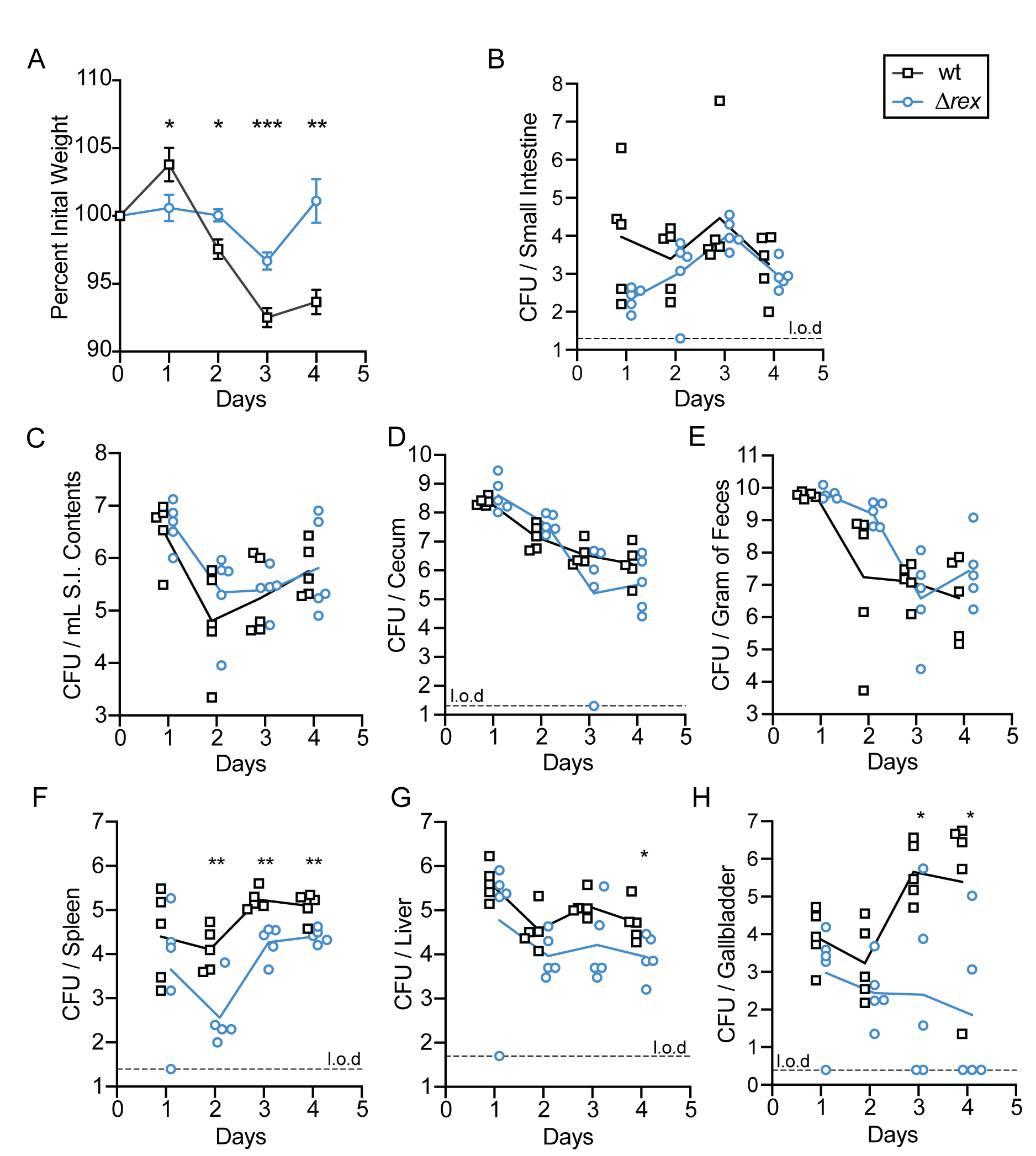

Supplement: S4 Fig — Female BALB/c mice were orally infected with 108 CFU of wt (black squares) or Δrex (blue circles) and the number of bacteria present in each tissue was determined over time. A. The body weights of the mice over time, reported as a percentage of body weight prior to infection. Data are the means and SEMs of n = 20 (day 1), n = 15 (day 2), n = 10 (day 3) and n = 5 (day 4). B-H. Mice were sacrificed each day and organs were harvested to enumerate bacterial burden. Each symbol represents an individual mouse (n = 5 per group), and the solid lines indicate the geometric means. Dashed lines indicate the limit of detection (l.o.d.). Results are expressed as log-transformed CFU per organ or per gram of feces. p values were calculated using a heteroscedastic Student’s t test. * p < 0.05; ** p < 0.01; *** p < 0.001. (TIF) [file ppat.1009379.s004.tif]

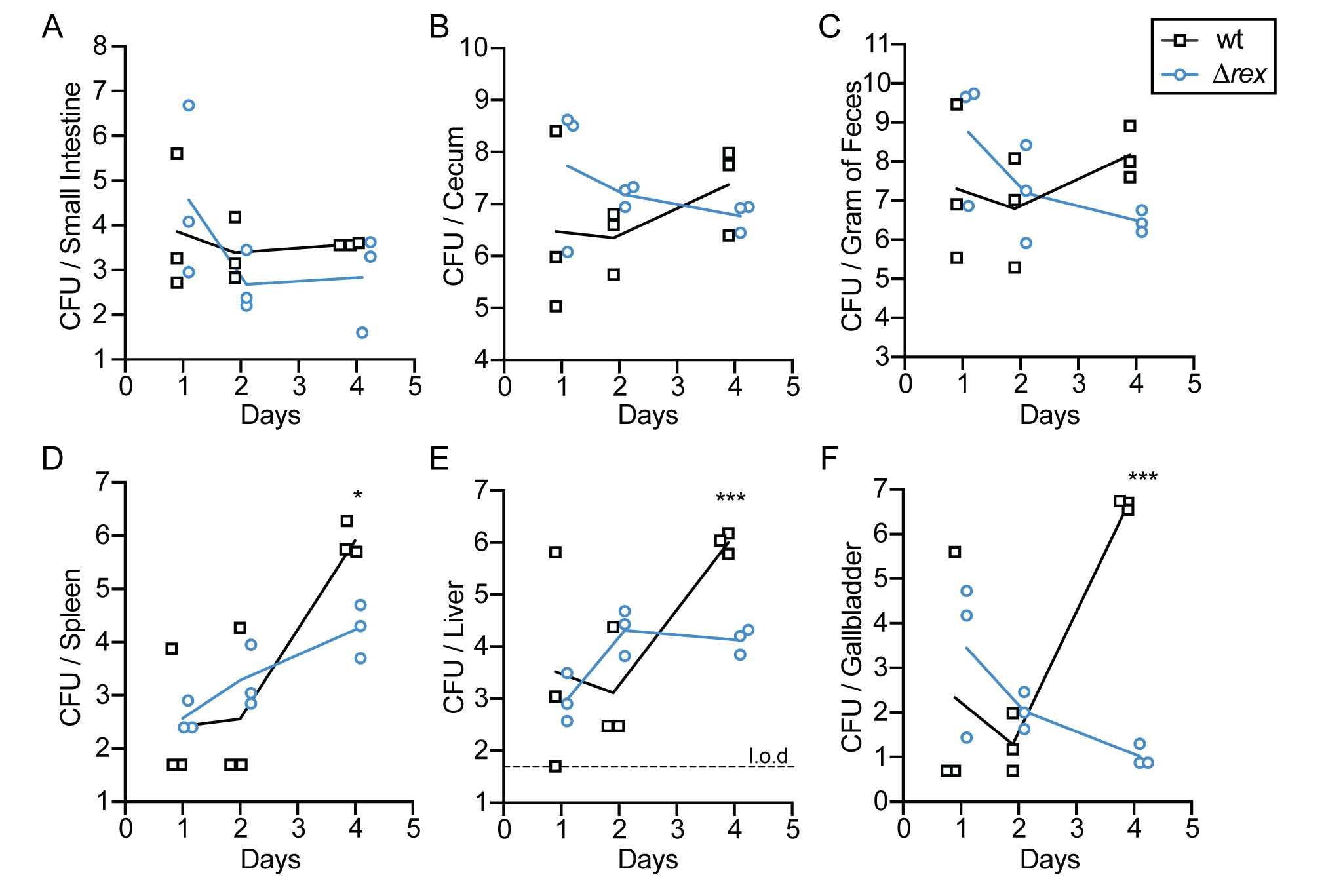

Supplement: S5 Fig — Female BALB/c mice were orally infected with 108 CFU of wt (black squares) or Δrex (blue circles) and the number of bacteria present in each tissue was determined over time. A-F. Mice were sacrificed on 1, 2, and 4 days post-infection and organs were harvested to enumerate bacterial burden. Panel A includes small intestinal tissue only; bacterial burden in the intestinal contents was not evaluated. Each symbol represents an individual mouse (n = 3 per group) and the solid lines indicate the geometric means. Dashed lines indicate the limit of detection (l.o.d.). Results are expressed as log-transformed CFU per organ or per gram of feces. p values were calculated using a heteroscedastic Student’s t test. * p < 0.05; *** p < 0.001. (TIF) [file ppat.1009379.s005.tif]
