# Supplementary material for: The redox-responsive transcriptional regulator Rex represses fermentative metabolism and is required for Listeria monocytogenes pathogenesis
Source: PLoS Pathog. 2021 Aug 16;17(8):e1009379. doi: 10.1371/journal.ppat.1009379 (PMC8389512; doi:10.1371/journal.ppat.1009379)
Supplement: S1 Text — Table A in S1 Text. All Rex repressed genes during stationary phase. Table B in S1 Text All Rex repressed genes during mid-log phase. Table C in S1 Text All transcripts less abundant in Δrex during stationary phase. Table D in S1 Text All transcripts less abundant in Δrex during mid-log phase. Table E in S1 Text Predicted Rex binding sites in the 10403S genome. Table F in S1 Text L. monocytogenes strains used in this study. Table G in S1 Text E. coli strains used in this study. (DOCX) [file ppat.1009379.s006.docx]

**SUPPORTING INFORMATION**

**The redox-responsive transcriptional regulator Rex represses fermentative metabolism and is required for *Listeria monocytogenes* pathogenesis**

Cortney R. Halsey^1^, Rochelle C. Glover^1^, Maureen K. Thomason^1^, Michelle L. Reniere^1^*

Department of Microbiology, University of Washington School of Medicine, Seattle, Washington, USA.

*reniere@uw.edu

**CONTENTS**

Table A. All Rex-repressed genes during stationary phase

Table B. All Rex-repressed genes during mid-log phase

Table C. All transcripts less abundant in ∆*rex* during stationary phase

Table D. All transcripts less abundant in ∆*rex* during mid-log phase

Table E. Predicted Rex binding sites in the 10403S genome

Table F. *L. monocytogenes* strains used in this study

Table G. *E. coli* strains used in this study

References

**Table A. All Rex repressed genes during stationary phase**

| **10403S** | **EGD-e** | **Gene** | **Function** | **Fold change in ∆*rex*** |
| --- | --- | --- | --- | --- |
| LMRG_01332 | lmo1634 | lap | bifunctional acetaldehyde-CoA/alcohol dehydrogenase | 342.30 |
| LMRG_00859 | lmo1407 | pflC | pyruvate formate-lyase 1-activating enzyme | 88.30 |
| LMRG_00858 | lmo1406 | pflB | formate acetyltransferase | 59.84 |
| LMRG_00046 | lmo0355 | - | fumarate reductase flavoprotein subunit | 85.24 |
| LMRG_01064 | lmo1917 | pflA | formate acetyltransferase | 77.91 |
| LMRG_00476 | lmo0788 | - | 2-hydroxyglutaryl-CoA dehydratase | 54.31 |
| LMRG_00573 | lmo1131 | - | ABC transporter | 41.97 |
| LMRG_00574 | lmo1132 | - | ABC transporter | 29.20 |
| LMRG_00566 | lmo1124 | - | hypothetical protein | 31.62 |
| LMRG_00564 | lmo1122 | - | hypothetical protein | 21.68 |
| LMRG_02862 | lmo1120 | - | hypothetical protein | 19.72 |
| LMRG_00563 | lmo1121 | - | hypothetical protein | 12.52 |
| LMRG_00565 | lmo1123 | - | hypothetical protein | 10.47 |
| LMRG_00568 | lmo1126 | - | hypothetical protein | 5.40 |
| LMRG_00569 | lmo1127 | - | hypothetical protein | 4.52 |
| LMRG_00567 | lmo1125 | - | hypothetical protein | 4.47 |
| LMRG_01259 | - | - | ferrous iron transport protein B | 31.58 |
| LMRG_01258 | lmo2105 | feoB | ferrous iron transporter B | 26.91 |
| LMRG_01257 | lmo2104 | feoA | ferrous iron transporter A | 18.26 |
| LMRG_02861 | lmo1297 | - | aluminum resistance protein | 26.23 |
| LMRG_01595 | lmo2237 | - | MFS domain-containing protein | 23.31 |
| LMRG_01594 | lmo2238 | - | MFS domain-containing protein | 15.18 |
| LMRG_01598 | lmo2234 | - | hypothetical protein | 9.93 |
| LMRG_01596 | lmo2236 | - | shikimate 5-dehydrogenase | 8.48 |
| LMRG_01597 | lmo2235 | - | NADH oxidase | 6.79 |
| LMRG_01945 | lmo2751 | - | ABC transporter | 21.37 |
| LMRG_01944 | lmo2752 | - | ABC transporter | 19.77 |
| LMRG_01645 | lmo2187 | - | hypothetical protein | 19.86 |
| LMRG_01979 | lmo2717 | cydB | cytochrome d ubiquinol oxidase subunit II | 19.05 |
| LMRG_01980 | lmo2716 | cydC | ABC transporter CydDC cysteine exporter CydD | 17.62 |
| LMRG_01981 | lmo2715 | cydD | ABC transporter CydDC cysteine exporter CydC | 15.76 |
| LMRG_01978 | lmo2718 | cydA | cytochrome bd-I oxidase subunit I | 13.99 |
| LMRG_01659 | lmo2173 | - | sigma-54-dependent transcriptional regulator, nitrogen fixation/regulation | 16.62 |
| LMRG_02863 | lmo1120 | - | hypothetical protein | 14.59 |
| LMRG_01838 | lmo2410 | - | hypothetical protein | 14.33 |
| LMRG_00104 | lmo0412 | - | hypothetical protein | 14.17 |
| LMRG_00561 | lmo1117 | - | VOC domain-containing protein | 13.99 |
| LMRG_02864 | lmo1120 | - | hypothetical protein | 13.88 |
| LMRG_01647 | lmo2185 | hbp2 | hypothetical protein | 13.37 |
| LMRG_01649 | lmo2183 | isdE | iron complex transport system permease | 8.68 |
| LMRG_01650 | lmo2182 | isdF | iron complex transport system ATP-binding protein | 8.05 |
| LMRG_01646 | lmo2186 | hbp1 | heme uptake protein IsdC | 6.61 |
| LMRG_01651 | lmo2181 | srtB | SrtB family sortase | 6.52 |
| LMRG_01648 | lmo2184 | isdD | heme ABC transporter heme-binding protein isdE | 6.43 |
| LMRG_01660 | lmo2172 | - | propionate CoA-transferase | 13.24 |
| LMRG_01661 | lmo2171 | - | major facilitator family transporter | 4.75 |
| LMRG_00127 | lmo0434 | inlB | internalin B | 10.97 |
| LMRG_00126 | lmo0433 | inlA | internalin A | 10.26 |
| LMRG_01801 | lmo2447 | - | Rgg/GadR/MutR family transcriptional regulator | 10.76 |
| LMRG_02012 | lmo0912 | - | formate transporter | 10.71 |
| LMRG_02060 | lmo0961 | - | protease | 10.66 |
| LMRG_02059 | lmo0960 | - | hypothetical protein | 7.84 |
| LMRG_02187 | lmo2642 | - | serine/threonine protein phosphatase | 10.39 |
| LMRG_02214 | lmo2669 | - | hypothetical protein | 9.90 |
| LMRG_02190 | lmo2644 | - | SMI1_KNR4 domain-containing protein | 9.89 |
| LMRG_02488 | lmo0058 | essA | ESAT-6 secretion machinery protein | 9.49 |
| LMRG_02487 | lmo0057 | esaA | polytopic membrane protein with 5 predicted transmembrane helices | 6.78 |
| LMRG_02486 | lmo0056 | esxA | ESAT-6-like protein | 4.99 |
| LMRG_02490 | lmo0060 | essB | YukC protein | 3.17 |
| LMRG_02491 | lmo0061 | essC | Integral membrane FtsK/SpoIIIE-type ATPase | 3.59 |
| LMRG_02193 | lmo2648 | - | hypothetical protein | 9.37 |
| LMRG_02192 | lmo2647 | - | creatinine amidohydrolase | 9.29 |
| LMRG_02195 | lmo2650 | - | PTS EIIB type-2 domain-containingl protein | 7.61 |
| LMRG_02194 | lmo2649 | ulaA | ascorbate-specific PTS system IIC component | 6.90 |
| LMRG_02191 | lmo2646 | - | hypothetical protein | 7.59 |
| LMRG_02196 | lmo2651 | - | mannitol-specific PTS system IIA component | 5.31 |
| LMRG_00171 | lmo0490 | - | shikimate 5-dehydrogenase | 8.79 |
| LMRG_00172 | lmo0491 | - | 3-dehydroquinate dehydratase type I | 2.34 |
| LMRG_01106 | lmo1959 | fhuD | iron complex transport system substrate-binding protein | 8.50 |
| LMRG_01105 | lmo1958 | - | iron complex transport system permease | 2.51 |
| LMRG_01104 | lmo1957 | - | iron complex transport system permease | 2.69 |
| LMRG_02138 | - | - | type II CRISPR-associated endonuclease Cas1 | 8.40 |
| LMRG_02137 | - | - | type II CRISPR RNA-guided endonuclease Cas9 | 8.16 |
| LMRG_02136 | - | - | CRISPR/Cas system-associated protein Cas2 | 7.64 |
| LMRG_01977 | lmo2719 | - | tRNA-adenosine deaminase | 5.36 |
| LMRG_02134 | lmo2594 | - | hypothetical protein | 4.50 |
| LMRG_02135 | lmo2594 | - | hypothetical protein | 3.07 |
| LMRG_01781 | lmo2467 | - | chitin-binding protein | 7.87 |
| LMRG_00865 | lmo1413 | - | peptidoglycan bound protein | 7.35 |
| LMRG_01652 | lmo2180 | - | hypothetical protein | 6.94 |
| LMRG_00223 | lmo0541 | - | iron complex transport system substrate-binding protein | 6.90 |
| LMRG_02783 | lmo0814 | - | enoyl-[acyl carrier protein] reductase II | 6.12 |
| LMRG_00760 | lmo1310 | - | PAPS_reduct domain-containing protein | 5.71 |
| LMRG_00757 | lmo1307 | - | hypothetical protein | 3.85 |
| LMRG_00758 | lmo1308 | - | Methyltransf_11 domain-containing protein | 3.77 |
| LMRG_00758 | lmo1309 | - | ParB domain-containing protein | 3.64 |
| LMRG_00761 | lmo1311 | - | Helicase ATP-binding domain-containing protein | 3.59 |
| LMRG_00155 | lmo0474 | - | hypothetical protein | 5.63 |
| LMRG_01217 | lmo2067 | bsh | bile acid hydrolase | 5.57 |
| LMRG_01621 | lmo2211 | hemH | ferrochelatase | 5.52 |
| LMRG_01620 | lmo2212 | hemE | uroporphyrinogen decarboxylase | 5.06 |
| LMRG_02595 | lmo0280 | - | highly anaerobic ribonucleotide reductase | 5.19 |
| LMRG_02596 | lmo0279 | - | anaerobic ribonucleoside triphosphate reductase | 4.13 |
| LMRG_00706 | lmo1257 | - | hypothetical protein | 5.16 |
| LMRG_02232 | lmo2686 | - | DUF5626 domain-containing protein | 5.11 |
| LMRG_02234 | lmo2688 | - | cell division protein FtsW/RodA/SpoVE | 2.18 |
| LMRG_02233 | lmo2687 | - | hypothetical protein | 2.41 |
| LMRG_02107 | lmo1007 | - | hypothetical protein | 5.02 |
| LMRG_01681 | lmo2151 | - | hypothetical protein | 4.97 |
| LMRG_00154 | lmo0458 | - | hypothetical protein | 4.96 |
| LMRG_00528 | lmo1066 | - | myo-inositol-1(or 4)-monophosphatase | 4.86 |
| LMRG_01662 | lmo2170 | - | enoyl-[acyl carrier protein] reductase II | 4.71 |
| LMRG_00861 | lmo1409 | - | multidrug efflux transporter | 4.62 |
| LMRG_00166 | lmo0485 | - | nitroreductase domain-containing protein | 4.27 |
| LMRG_00005 | lmo0308 | - | hypothetical protein | 4.22 |
| LMRG_00059 | lmo0367 | fepB | Ferrous iron transport peroxidase | 4.16 |
| LMRG_00058 | lmo0366 | - | Lipoprotein | 3.26 |
| LMRG_02521 | lmo1750 | - | hypothetical protein | 4.01 |
| LMRG_02262 | lmo0839 | - | tetracycline resistance protein | 3.95 |
| LMRG_01107 | lmo1960 | fhuC | ferrichrome ABC transporter ATP-binding protein | 3.70 |
| LMRG_01108 | lmo1961 | - | ferredoxin-NADP reductase 1 | 3.02 |
| LMRG_01814 | lmo2434 | gadD3 | glutamate decarboxylase | 3.63 |
| LMRG_02908 | lmo2610 | - | translation initiation factor IF-1 | 3.58 |
| LMRG_01092 | lmo1945 | - | riboflavin transporter | 3.47 |
| LMRG_00134 | lmo0442 | - | hypothetical protein | 3.46 |
| LMRG_01415 | lmo1553 | hemL | glutamate-1-semialdehyde-2,1-aminomutase | 3.46 |
| LMRG_01412 | lmo1556 | hemC | porphobilinogen deaminase | 2.21 |
| LMRG_01413 | lmo1555 | hemD | uroporphyrinogen-III synthase | 2.77 |
| LMRG_01414 | lmo1554 | - | delta-aminolevulinic acid dehydratase | 2.83 |
| LMRG_02632 | lmo0210 | ldhA | lactate dehydrogenase | 3.30 |
| LMRG_02458 | lmo0029 | - | DUF4064 domain-containing protein | 3.12 |
| LMRG_02385 | lmo0136 | - | oligopeptide ABC transport | 3.07 |
| LMRG_02384 | lmo0135 | - | oligopeptide ABC transport | 2.52 |
| LMRG_02386 | lmo0137 | - | oligopeptide ABC transport | 2.75 |
| LMRG_02822 | lmo1798 | - | hypothetical protein | 3.06 |
| LMRG_02823 | lmo1799 | - | gram-pos-anchoring domain-containing protein | 2.23 |
| LMRG_01377 | lmo1590 | argJ | bifunctional ornithine acetyltransferase/N-acetylglutamate synthase | 3.03 |
| LMRG_01380 | lmo1587 | argF | ornithine carbamoyltransferase | 2.18 |
| LMRG_01911 | lmo2786 | - | ADP-ribosylglycohydrolase | 2.94 |
| LMRG_00642 | lmo1196 | - | precorrin-8W decarboxylase | 2.94 |
| LMRG_01453 | lmo1517 | glnK | nitrogen regulatory protein P-II | 2.92 |
| LMRG_01267 | lmo2113 | - | heme peroxidase | 2.90 |
| LMRG_01140 | lmo1992 | - | alpha-acetolactate decarboxylase | 2.85 |
| LMRG_00613 | lmo1170 | - | GHMP kinases N domain-containing protein | 2.82 |
| LMRG_00895 | lmo1443 | - | Branched-chain amino acid transport protein | 2.78 |
| LMRG_00894 | lmo1442 | - | hypothetical protein | 2.38 |
| LMRG_00893 | lmo1441 | - | 4-hydroxy-3-methylbut-2-en-1-yl diphosphate synthase | 2.27 |
| LMRG_01495 | lmo2348 | - | polar amino acid transport system permease | 2.74 |
| LMRG_01493 | lmo2350 | - | N-acetyltransferase domain-containing protein | 2.43 |
| LMRG_01494 | lmo2349 | - | polar amino acid transport system substrate-binding protein | 2.04 |
| LMRG_01499 | lmo2344 | - | glutaredoxin domain-containing protein | 2.25 |
| LMRG_02716 | lmo2371 | - | FtsX domain-containing protein | 2.73 |
| LMRG_02717 | lmo2372 | - | ABC trainsporter domain-containing protein | 2.65 |
| LMRG_01154 | lmo2006 | alsS | acetolactate synthase catabolic | 2.72 |
| LMRG_00021 | lmo0327 | - | internalin | 2.69 |
| LMRG_00644 | lmo1198 | - | cobalamin biosynthesis protein CbiG | 2.65 |
| LMRG_01544 | lmo2288 | - | gp15 | 2.65 |
| LMRG_02347 | lmo0098 | - | PTS mannose transporter subunit IID | 2.62 |
| LMRG_00004 | lmo0307 | - | hypothetical protein | 2.61 |
| LMRG_02003 | lmo0903 | - | OsmC/Ohr family protein | 2.59 |
| LMRG_00632 | lmo1186 | - | ethanolamine transporter | 2.52 |
| LMRG_00610 | lmo1167 | - | glycerol uptake facilitator protein | 2.51 |
| LMRG_02397 | lmo0152 | - | peptide ABC transporter substrate-binding protein | 2.51 |
| LMRG_01172 | lmo2023 | - | L-aspartate oxidase | 2.50 |
| LMRG_01173 | lmo2024 | - | nicotinate-nucleotide diphosphorylase | 2.00 |
| LMRG_01815 | lmo2433 | - | tributyrin esterase | 2.49 |
| LMRG_01222 | lmo2071 | - | hypothetical protein | 2.45 |
| LMRG_01221 | lmo2070 | - | hypothetical protein | 2.16 |
| LMRG_02016 | lmo0916 | - | cellobiose-specific PTS system IIA component | 2.43 |
| LMRG_02014 | lmo0914 | - | cellobiose-specific PTS system IIB component | 2.18 |
| LMRG_02017 | lmo0917 | - | beta-glucosidase | 2.11 |
| LMRG_00743 | lmo1293 | glpD | glycerol-3-phosphate dehydrogenase | 2.42 |
| LMRG_00607 | lmo1164 | - | ATP:Cob(I)alamin adenosyltransferase | 2.38 |
| LMRG_00169 | lmo0488 | - | LysR family transcriptional regulator | 2.34 |
| LMRG_00226 | lmo0544 | - | glucitol/sorbitol-specific PTS system IIC component | 2.33 |
| LMRG_01872 | lmo2826 | - | efflux protein, MFS family | 2.32 |
| LMRG_00691 | lmo1245 | - | hypothetical protein | 2.32 |
| LMRG_00609 | lmo1166 | pduQ | propanol dehydrogenase PduQ | 2.29 |
| LMRG_00601 | lmo1158 | - | BMC domain-containing protein | 2.29 |
| LMRG_00045 | lmo0354 | - | long-chain fatty-acid-CoA ligase | 2.28 |
| LMRG_00196 | lmo0515 | - | universal stress protein | 2.27 |
| LMRG_00372 | lmo0684 | - | hypothetical protein | 2.26 |
| LMRG_00332 | lmo0645 | - | APA family basic amino acid/polyamine antiporter | 2.26 |
| LMRG_00645 | lmo1199 | - | precorrin-3B C17-methyltransferase | 2.25 |
| LMRG_02321 | lmo0897 | - | SulP family sulfate permease | 2.23 |
| LMRG_01491 | lmo2352 | - | HTH-type transcriptional regulator ytlI | 2.21 |
| LMRG_00606 | lmo1163 | - | ethanolamine and carbon dioxide metabolism | 2.18 |
| LMRG_00176 | lmo0495 | - | hypothetical protein | 2.15 |
| LMRG_01213 | lmo2063 | - | hypothetical protein | 2.13 |
| LMRG_00333 | lmo0646 | - | hypothetical protein | 2.13 |
| LMRG_00066 | lmo0374 | - | cellobiose-specific phosphotransferase enzyme IIB component | 2.11 |
| LMRG_02114 | lmo1014 | - | glycine betaine/proline transport system ATP-binding protein | 2.11 |
| LMRG_02116 | lmo1016 | - | glycine betaine-binding protein | 2.00 |
| LMRG_00518 | lmo1056 | - | hypothetical protein | 2.11 |
| LMRG_00322 | lmo0637 | - | UbiE/COQ5 family methyltransferase | 2.09 |
| LMRG_00225 | lmo0543 | - | PTS EIIB type-5 domain-containing protein | 2.08 |
| LMRG_00619 | lmo1173 | - | sensor histidine kinase | 2.07 |
| LMRG_01014 | lmo1867 | - | pyruvate, phosphate dikinase | 2.07 |
| LMRG_01534 | lmo2298 | - | phage minor capsid protein | 2.06 |
| LMRG_00165 | lmo0484 | - | heme-degrading monooxygenase IsdG | 2.05 |
| LMRG_01880 | lmo2818 | - | MFS transporter | 2.02 |
| LMRG_00896 | lmo1444 | - | foldase PrsA | 2.01 |
| LMRG_00200 | lmo0519 | - | lincomycin resistance protein lmrB | 2.01 |
| LMRG_00604 | lmo1161 | eutJ | ethanolamine utilization protein EutJ | 2.01 |
| LMRG_02252 | lmo0829 | porA | pyruvate:ferredoxin oxidoreductase | 2.01 |
| LMRG_00153 | lmo0458 | - | hypothetical protein | 2.00 |

Highlighted genes are predicted to be in an operon [1].

**Table B. All Rex repressed genes during mid-log phase**

| **10403S** | **EGD-e** | **Gene** | **Function** | **Fold change in ∆*rex*** |
| --- | --- | --- | --- | --- |
| LMRG_00046 | lmo0355 | frdA | fumarate reductase flavoprotein subunit | 12.32 |
| LMRG_01945 | lmo2751 | - | ABC transporter | 12.03 |
| LMRG_01944 | lmo2752 | - | ABC transporter | 11.52 |
| LMRG_01660 | lmo2172 | - | propionate CoA-transferase | 10.84 |
| LMRG_01661 | lmo2171 | - | major facilitator family transporter | 7.18 |
| LMRG_02489 | lmo0059 | esaB | YukD protein | 10.13 |
| LMRG_02487 | lmo0057 | esaA | polytopic membrane protein with 5 predicted transmembrane helices | 5.99 |
| LMRG_02486 | lmo0056 | esxA | ESAT-6-like protein substrate | 5.85 |
| LMRG_02488 | lmo0058 | essA | ESAT-6 secretion machinery protein | 5.40 |
| LMRG_02490 | lmo0060 | essB | YukC protein | 5.04 |
| LMRG_02491 | lmo0061 | essC | FtsK/SpoIIIE-type ATPase | 3.82 |
| LMRG_02492 | - | - | FtsK/SpoIIIE-type ATPase | 3.69 |
| LMRG_02494 | - | - | FtsK/SpoIIIE-type ATPase | 2.65 |
| LMRG_01332 | lmo1634 | lap | bifunctional acetaldehyde-CoA/alcohol dehydrogenase | 7.10 |
| LMRG_01781 | lmo2467 | - | chitin-binding protein | 6.89 |
| LMRG_02595 | lmo0280 | - | highly anaerobic ribonucleotide reductase | 6.58 |
| LMRG_02596 | lmo0279 | - | anaerobic ribonucleoside triphosphate reductase | 5.57 |
| LMRG_00859 | lmo1407 | pflC | pyruvate formate-lyase 1-activating enzyme | 5.91 |
| LMRG_00858 | lmo1406 | pflB | formate acetyltransferase | 3.58 |
| LMRG_02861 | lmo1297 | - | aluminum resistance protein | 5.07 |
| LMRG_01595 | lmo2237 | - | MFS domain-containing protein | 5.02 |
| LMRG_01596 | lmo2236 | - | shikimate 5-dehydrogenase | 4.99 |
| LMRG_01594 | lmo2238 | - | MFS domain-containing protein | 4.79 |
| LMRG_01597 | lmo2235 | - | NADH oxidase | 3.09 |
| LMRG_01598 | lmo2234 | - | AP-endonuc-2 domain-containing protein | 2.05 |
| LMRG_02136 | - | - | CRISPR/Cas system-associated protein Cas2 | 5.00 |
| LMRG_02134 | - | - | hypothetical protein | 4.83 |
| LMRG_02137 | - | - | hypothetical protein | 4.20 |
| LMRG_02135 | - | - | hypothetical protein | 3.58 |
| LMRG_02138 | - | - | hypothetical protein | 2.29 |
| LMRG_01801 | lmo2447 | - | HTH cro/C1-type domain-containing protein | 4.95 |
| LMRG_00574 | lmo1132 | - | ABC transporter | 4.89 |
| LMRG_00573 | lmo1131 | - | ABC transporter | 4.14 |
| LMRG_00476 | lmo0788 | - | hypothetical protein | 4.66 |
| LMRG_02908 | lmo2610 | - | translation initiation factor IF-1 | 4.59 |
| LMRG_00321 | lmo0637 | - | UbiE/COQ5 family methyltransferase | 4.41 |
| LMRG_01645 | lmo2187 | - | hypothetical protein | 4.15 |
| LMRG_01681 | lmo2151 | - | hypothetical protein | 4.13 |
| LMRG_02012 | lmo0912 | - | formate transporter | 4.05 |
| LMRG_00564 | lmo1122 | - | hypothetical protein | 3.95 |
| LMRG_00566 | lmo1124 | - | hypothetical protein | 3.86 |
| LMRG_02862 | lmo1120 | - | hypothetical protein | 3.55 |
| LMRG_02863 | lmo1120 | - | hypothetical protein | 3.06 |
| LMRG_00565 | lmo1123 | - | hypothetical protein | 2.80 |
| LMRG_00568 | lmo1126 | - | Acetyltransferase | 2.76 |
| LMRG_02864 | lmo1120 | - | hypothetical protein | 2.73 |
| LMRG_00567 | lmo1125 | - | hypothetical protein | 2.47 |
| LMRG_00563 | lmo1121 | - | hypothetical protein | 2.41 |
| LMRG_00569 | lmo1127 | - | hypothetical protein | 2.34 |
| LMRG_01259 | - | - | ferrous iron transport protein B | 3.69 |
| LMRG_01258 | lmo2105 | - | ferrous iron transporter B | 3.15 |
| LMRG_01257 | lmo2104 | - | ferrous iron transporter A | 3.00 |
| LMRG_01064 | lmo1917 | pflA | formate acetyltransferase | 3.58 |
| LMRG_00228 | lmo0546 | - | sorbitol-6-phosphate 2-dehydrogenase | 3.53 |
| LMRG_00760 | lmo1310 | - | PAPS-reduct domain-containing protein | 3.23 |
| LMRG_00759 | lmo1309 | - | ParB domain-containing protein | 2.12 |
| LMRG_00758 | lmo1308 | - | Methyltransf-11 domain-containing protein | 2.01 |
| LMRG_00761 | lmo1311 | - | Helicase ATP-binding domain-containing protein | 2.89 |
| LMRG_00762 | lmo1312 | - | hypothetical protein | 2.39 |
| LMRG_02060 | lmo0961 | - | protease | 3.08 |
| LMRG_02059 | lmo0960 | - | hypothetical protein | 2.43 |
| LMRG_01662 | lmo2170 | - | enoyl-[acyl carrier protein] reductase II | 2.99 |
| LMRG_02214 | lmo2669 | - | hypothetical protein | 2.88 |
| LMRG_00561 | lmo1117 | - | VOC domain-containing protein | 2.87 |
| LMRG_02233 | lmo2687 | - | hypothetical protein | 2.84 |
| LMRG_02234 | lmo2688 | - | cell division protein FtsW/RodA/SpoVE | 2.79 |
| LMRG_02235 | lmo2689 | - | magnesium-translocating P-type ATPase | 2.40 |
| LMRG_02232 | lmo2686 | - | DUF5626 domain-containing protein | 2.33 |
| LMRG_02632 | lmo0210 | ldhA | lactate dehydrogenase | 2.81 |
| LMRG_00227 | lmo0545 | - | glucitol operon activator protein | 2.77 |
| LMRG_00748 | lmo1298 | glnR | nitrogen regulatory protein | 2.77 |
| LMRG_00475 | lmo0787 | - | amino acid transporter AAT family protein | 2.76 |
| LMRG_01172 | lmo2023 | - | L-aspartate oxidase | 2.66 |
| LMRG_01174 | lmo2025 | - | quinolinate synthetase complex A subunit | 2.11 |
| LMRG_02850 | - | - | GntR family transcriptional regulator | 2.66 |
| LMRG_01542 | lmo2290 | - | gp13 | 2.62 |
| LMRG_01541 | lmo2291 | - | major tail shaft protein | 2.51 |
| LMRG_02187 | lmo2642 | - | serine/threonine protein phosphatase | 2.55 |
| LMRG_00127 | lmo0434 | - | internalin B | 2.54 |
| LMRG_01092 | lmo1945 | - | riboflavin transporter | 2.50 |
| LMRG_02321 | lmo0897 | - | SulP family sulfate permease | 2.40 |
| LMRG_00171 | lmo0490 | - | shikimate 5-dehydrogenase | 2.38 |
| LMRG_01977 | lmo2719 | - | tRNA-adenosine deaminase | 2.34 |
| LMRG_01979 | lmo2717 | cydB | cytochrome d ubiquinol oxidase subunit II | 2.32 |
| LMRG_01981 | lmo2715 | cydD | ABC transporter CydDC cysteine exporter CydC | 2.21 |
| LMRG_01980 | lmo2716 | cydC | ABC transporter CydDC cysteine exporter CydD | 2.14 |
| LMRG_01978 | lmo2718 | cydA | cytochrome bd-I oxidase subunit I | 2.02 |
| LMRG_02458 | lmo0029 | - | DUF4064 domain-containing protein | 2.27 |
| LMRG_00691 | lmo1245 | - | hypothetical protein | 2.21 |
| LMRG_00104 | lmo0412 | - | hypothetical protein | 2.16 |
| LMRG_00435 | lmo0747 | - | hypothetical protein | 2.14 |
| LMRG_00275 | lmo0593 | - | formate/nitrite transporter | 2.12 |
| LMRG_02849 | - | - | GntR family transcriptional regulator | 2.05 |
| LMRG_01813 | lmo2435 | - | hypothetical protein | 2.02 |
| LMRG_02846 | - | - | GntR family transcriptional regulator | 2.02 |
| LMRG_01621 | lmo2211 | - | ferrochelatase | 2.02 |

Highlighted genes are predicted to be in an operon [1].

**Table C. All transcripts less abundant in ∆*rex* during stationary phase**

| **10403S** | **EGD-e** | **Gene** | **Function** | **Fold change in ∆*rex*** |
| --- | --- | --- | --- | --- |
| LMRG_02208 | lmo2663 | - | L-iditol 2-dehydrogenase | -59.24 |
| LMRG_02209 | lmo2664 | - | L-iditol 2-dehydrogenase | -44.03 |
| LMRG_02210 | lmo2665 | - | galactitol-specific PTS system IIC component | -24.78 |
| LMRG_02211 | lmo2666 | - | galactitol-specific PTS system IIB component | -18.37 |
| LMRG_02212 | lmo2667 | - | PTS EIIA type-2 domain-containing protein | -14.41 |
| LMRG_02213 | lmo2668 | - | hypothetical protein | -12.90 |
| LMRG_02207 | lmo2662 | - | ribose 5-phosphate isomerase B | -6.25 |
| LMRG_02206 | lmo2661 | - | ribulose-phosphate 3-epimerase | -5.79 |
| LMRG_02205 | lmo2660 | - | transketolase | -3.33 |
| LMRG_02204 | lmo2659 | - | ribulose-phosphate 3-epimerase | -2.81 |
| LMRG_01934 | lmo2761 | - | beta-glucosidase | -14.37 |
| LMRG_01933 | lmo2762 | - | cellobiose-specific PTS system IIB component | -11.50 |
| LMRG_01931 | lmo2764 | - | hypothetical protein | -7.68 |
| LMRG_01932 | lmo2763 | - | cellobiose-specific PTS system IIC component | -7.54 |
| LMRG_01930 | lmo2765 | - | cellobiose-specific PTS system IIA component | -7.43 |
| LMRG_02304 | lmo0880 | - | peptidoglycan bound protein | -9.71 |
| LMRG_05015 | lmo2824 | - | D-3-phosphoglycerate dehydrogenase | -9.37 |
| LMRG_01669 | lmo2163 | - | hypothetical protein | -8.41 |
| LMRG_01673 | lmo2159 | - | hypothetical protein | -7.10 |
| LMRG_01670 | lmo2162 | - | AP-endonuc-2 domain-containing protein | -7.12 |
| LMRG_01671 | lmo2161 | - | ThuA domain-containing protein | -6.86 |
| LMRG_01672 | lmo2160 | - | sugar phosphate isomerase/epimerase | -6.05 |
| LMRG_01943 | lmo2753 | - | hypothetical protein | -7.24 |
| LMRG_00094 | lmo0401 | - | alpha-mannosidase | -4.89 |
| LMRG_00093 | lmo0400 | - | fructose-specific PTS system IIC component | -4.52 |
| LMRG_00091 | lmo0398 | - | fructose-specific PTS system IIA component | -4.33 |
| LMRG_00092 | lmo0399 | - | fructose-specific PTS system IIB component | -3.90 |
| LMRG_00095 | lmo0402 | - | PRD/PTS system IIA 2 domain-containing protein | -3.71 |
| LMRG_01725 | lmo2523 | - | single-strand binding protein family | -4.68 |
| LMRG_02681 | lmo2586 | fdh | formate dehydrogenase alpha subunit | -3.88 |
| LMRG_02682 | lmo2585 | - | hypothetical protein | -3.58 |
| LMRG_01889 | lmo2809 | - | hypothetical protein | -3.85 |
| LMRG_01677 | lmo2155 | - | ribonucleoside-diphosphate reductase subunit alpha | -3.80 |
| LMRG_01680 | lmo2152 | - | thioredoxin | -3.43 |
| LMRG_01678 | lmo2154 | - | ribonucleoside-diphosphate reductase | -3.26 |
| LMRG_01679 | lmo2153 | - | ribonucleotide reductase-associated flavodoxin | -2.98 |
| LMRG_01674 | lmo2158 | - | CsbD domain-containing protein | -3.66 |
| LMRG_00042 | lmo0351 | - | dihydroxyacetone kinase | -3.64 |
| LMRG_00037 | lmo0346 | - | triosephosphate isomerase | -2.62 |
| LMRG_00041 | lmo0350 | - | hypothetical protein | -2.50 |
| LMRG_00039 | lmo0348 | - | dihydroxyacetone kinase | -2.44 |
| LMRG_00035 | lmo0344 | - | short chain dehydrogenase | -2.34 |
| LMRG_00034 | lmo0343 | - | transaldolase | -2.20 |
| LMRG_00038 | lmo0347 | - | dihydroxyacetone kinase L subunit | -2.16 |
| LMRG_00033 | lmo0342 | - | transketolase | -2.04 |
| LMRG_00036 | lmo0345 | - | ribose 5-phosphate isomerase B | -2.02 |
| LMRG_02700 | lmo2568 | - | hypothetical protein | -3.58 |
| LMRG_01726 | lmo2522 | - | hypothetical protein | -3.54 |
| LMRG_00327 | lmo0641 | frvA | cadmium-translocating P-type ATPase | -3.41 |
| LMRG_00798 | lmo1348 | gcvT | glycine cleavage system T protein | -3.39 |
| LMRG_00799 | lmo1349 | - | glycine cleavage system P-protein | -2.71 |
| LMRG_00800 | lmo1350 | - | glycine dehydrogenase subunit 2 | -2.48 |
| LMRG_00989 | lmo1842 | - | STAS domain-containing protein | -3.38 |
| LMRG_02395 | lmo0149 | - | hypothetical protein | -3.32 |
| LMRG_02683 | lmo2584 | fdhD | formate dehydrogenase family accessory protein | -3.17 |
| LMRG_00315 | lmo0632 | - | fructose-specific PTS system IIC component | -3.12 |
| LMRG_00479 | lmo0791 | - | DUF5105 domain-containing protein | -2.98 |
| LMRG_02982 | lmo0827 | - | transposase | -2.94 |
| LMRG_00011 | lmo0318 | - | thiamine-phosphate pyrophosphorylase | -2.91 |
| LMRG_00009 | lmo0316 | - | hydroxyethylthiazole kinase | -2.89 |
| LMRG_00008 | lmo0315 | - | thiamine biosynthesis protein | -2.62 |
| LMRG_00010 | lmo0317 | - | phosphomethylpyrimidine kinase | -2.48 |
| LMRG_01025 | lmo1878 | - | transcriptional regulator mntR | -2.89 |
| LMRG_00287 | lmo0604 | - | hypothetical protein | -2.86 |
| LMRG_02255 | lmo0832 | - | transposase | -2.77 |
| LMRG_01431 | lmo1539 | - | glycerol uptake facilitator protein | -2.74 |
| LMRG_02654 | lmo0255 | - | hypothetical protein | -2.72 |
| LMRG_02461 | lmo0032 | - | xylose repressor | -2.67 |
| LMRG_00026 | lmo0334 | - | Flavodoxin-5 domain-containingprotein | -2.62 |
| LMRG_02568 | lmo0300 | - | phospho-beta-galactosidase | -2.59 |
| LMRG_01806 | lmo2442 | - | DUF218 domain-containing protein | -2.59 |
| LMRG_02570 | lmo0298 | - | PTS beta-glucoside transporter subunit IIC | -2.55 |
| LMRG_02684 | lmo2583 | - | response regulator | -2.53 |
| LMRG_00183 | lmo0502 | - | SIS domain-containing protein | -2.45 |
| LMRG_00188 | lmo0507 | - | galactitol-specific PTS system IIB component | -2.25 |
| LMRG_00184 | lmo0503 | - | galactitol-specific PTS system IIA component | -2.19 |
| LMRG_00182 | lmo0501 | - | transcription antiterminator | -2.09 |
| LMRG_00868 | lmo1416 | - | VanZ domain-containing protein protein | -2.45 |
| LMRG_02567 | lmo0301 | - | PTS beta-glucoside transporter subunit IIA | -2.42 |
| LMRG_00330 | lmo0643 | - | transaldolase | -2.41 |
| LMRG_00087 | lmo0394 | - | extracellular P60 protein | -2.40 |
| LMRG_00305 | lmo0622 | - | hypothetical protein | -2.40 |
| LMRG_00453 | lmo0765 | - | hypothetical protein | -2.39 |
| LMRG_02967 | lmo0191 | - | phospho-beta-glucosidase | -2.37 |
| LMRG_01809 | lmo2439 | - | hypothetical protein | -2.35 |
| LMRG_00378 | lmo0690 | - | flagellin | -2.35 |
| LMRG_00498 | lmo1037 | - | hypothetical protein | -2.30 |
| LMRG_00158 | lmo0477 | - | secreted protein | -2.26 |
| LMRG_01657 | lmo2175 | - | 3-oxoacyl-[acyl-carrier protein] reductase | -2.26 |
| LMRG_01560 | lmo2270 | comK | competence protein | -2.20 |
| LMRG_01145 | lmo1997 | - | mannose-specific PTS system IIA component | -2.20 |
| LMRG_01146 | lmo1998 | - | sugar isomerase domain-containing protein | -2.13 |
| LMRG_00048 | lmo0357 | - | fructose-specific PTS system IIA component | -2.18 |
| LMRG_01279 | lmo2125 | - | maltose/maltodextrin transport system substrate-binding protein | -2.18 |
| LMRG_00096 | lmo0403 | - | DUF 4064 domain-containing protein | -2.17 |
| LMRG_02571 | lmo0297 | - | transcriptional antiterminator | -2.12 |
| LMRG_01628 | lmo2204 | - | hypothetical protein | -2.11 |
| LMRG_00881 | lmo1429 | - | proton-coupled thiamine transporter | -2.10 |
| LMRG_02456 | lmo0027 | - | beta-glucoside-specificPTS system IIABC component | -2.08 |
| LMRG_02701 | lmo2567 | - | hypothetical protein | -2.08 |
| LMRG_02770 | lmo1696 | - | VanZ domain-containing protein | -2.07 |
| LMRG_00860 | lmo1408 | - | PadR domain-containing protein | -2.06 |
| LMRG_00198 | lmo0517 | - | phosphoglycerate mutase | -2.05 |
| LMRG_01708 | lmo2539 | - | serine hydroxymethyltransferase | -2.05 |
| LMRG_00947 | lmo1800 | - | protein-tyrosine phosphatase | -2.04 |
| LMRG_01655 | lmo2177 | - | hypothetical protein | -2.04 |
| LMRG_02478 | lmo0049 | - | hypothetical protein | -2.04 |
| LMRG_01026 | lmo1879 | - | cold shock protein | -2.03 |
| LMRG_00191 | lmo0510 | - | hypothetical protein | -2.01 |
| LMRG_00588 | lmo1145 | eutP | ethanolamine utilization protein | -2.00 |

Highlighted genes are predicted to be in an operon [1].

**Table D. All transcripts less abundant in ∆*rex* during mid-log phase**

| **10403S** | **EGD-e** | **Gene** | **Function** | **Fold change in ∆*rex*** |
| --- | --- | --- | --- | --- |
| LMRG_02304 | lmo0880 | - | peptidoglycan bound protein | -6.75 |
| LMRG_01674 | lmo2158 | - | CsbD domain-containing protein | -6.30 |
| LMRG_00311 | lmo0628 | - | hypothetical protein | -4.06 |
| LMRG_01561 | lmo2269 | - | IDEAL domain-containing protein | -3.47 |
| LMRG_00230 | lmo0548 | - | hypothetical protein | -3.35 |
| LMRG_01619 | lmo2213 | - | ABM domain-containing protein | -3.23 |
| LMRG_02611 | lmo0265 | - | succinyl-diaminopimelate desuccinylase | -3.12 |
| LMRG_02732 | lmo2387 | - | hypothetical protein | -3.07 |
| LMRG_00341 | lmo0654 | - | hypothetical protein | -3.01 |
| LMRG_00687 | lmo1241 | - | hypothetical protein | -2.99 |
| LMRG_00009 | lmo0316 | - | hydroxyethylthiazole kinase | -2.84 |
| LMRG_00008 | lmo0315 | - | thiamine biosynthesis protein | -2.77 |
| LMRG_00010 | lmo0317 | - | phosphomethylpyrimidine kinase | -2.72 |
| LMRG_00011 | lmo0318 | - | thiamine-phosphate pyrophosphorylase | -2.63 |
| LMRG_00479 | lmo0791 | - | DUF5105 domain-containing protein | -2.80 |
| LMRG_00131 | lmo0439 | - | hypothetical protein | -2.78 |
| LMRG_02808 | lmo2132 | - | hypothetical protein | -2.73 |
| LMRG_02646 | lmo0263 | inlH | internalin H | -2.64 |
| LMRG_02052 | lmo0953 | - | hypothetical protein | -2.63 |
| LMRG_00293 | lmo0610 | - | internalin protein | -2.62 |
| LMRG_00013 | lmo0321 | - | hypothetical protein | -2.62 |
| LMRG_01948 | lmo2748 | - | general stress protein 26 | -2.54 |
| LMRG_02809 | lmo2131 | - | hypothetical protein | -2.54 |
| LMRG_01602 | lmo2230 | - | arsenate reductase | -2.46 |
| LMRG_00137 | lmo0445 | - | transcriptional regulator | -2.43 |
| LMRG_02094 | lmo0994 | - | DUF4064 domain-containing protein | -2.42 |
| LMRG_02768 | lmo1694 | - | CDP-abequose synthase | -2.38 |
| LMRG_01376 | lmo1591 | - | N-acetyl-gamma-glutamyl-phosphate reductase | -2.37 |
| LMRG_00327 | lmo0641 | - | cadmium-translocating P-type ATPase | -2.32 |
| LMRG_02393 | lmo0149 | - | hypothetical protein | -2.31 |
| LMRG_00285 | lmo0602 | - | N-acetyltransferase domain-containing protein | -2.29 |
| LMRG_00236 | lmo0554 | - | NADH-dependent butanol dehydrogenase | -2.27 |
| LMRG_02095 | lmo0995 | - | YkrP protein | -2.25 |
| LMRG_01669 | lmo2163 | - | oxidoreductase | -2.21 |
| LMRG_01241 | lmo2090 | - | argininosuccinate synthase | -2.21 |
| LMRG_00595 | lmo1152 | pduB | propanediol utilization protein | -2.21 |
| LMRG_01943 | lmo2753 | - | hypothetical protein | -2.20 |
| LMRG_01133 | lmo1985 | ilvH | acetolactate synthase small subunit | -2.19 |
| LMRG_00334 | lmo0647 | - | hypothetical protein | -2.16 |
| LMRG_00196 | lmo0515 | - | universal stress protein | -2.15 |
| LMRG_02218 | lmo2673 | - | universal stress protein | -2.14 |
| LMRG_01431 | lmo1539 | - | glycerol uptake facilitator protein | -2.12 |
| LMRG_02472 | lmo0043 | - | arginine deiminase | -2.11 |
| LMRG_02451 | lmo0022 | - | PTS fructose transporter subunit IIB | -2.09 |
| LMRG_02036 | lmo0937 | - | hypothetical protein | -2.09 |
| LMRG_00482 | lmo0794 | - | NAD(P)-bd-dom domain-containing protein | -2.07 |
| LMRG_02695 | lmo2573 | - | NADPH2:quinone reductase | -2.05 |
| LMRG_01601 | lmo2231 | - | AT-dimer domain-containing protein | -2.05 |
| LMRG_00590 | lmo1147 | copB | cobalamin biosynthesis protein | -2.04 |
| LMRG_00037 | lmo0346 | - | triosephosphate isomerase | -2.01 |

Highlighted genes are predicted to be in an operon [1].

**Table E. Predicted Rex binding sites in the 10403S genome**

| **LMRG** | **Gene** | **Function** | **ROP Sequence 5'-3' ^a^** | **Mismatches** |
| --- | --- | --- | --- | --- |
| LMRG_00196 | - | universal stress protein | ATTGTGAATTCATTAGCAAA | 0 |
| LMRG_00573 | - | ABC transporter | TTTGTGATATAATAAACAAA | 1 |
| LMRG_00574 | - | ABC transporter |  |  |
| LMRG_01661 | - | major facilitator family transporter | TATGTGAATAATTGAACAAT | 1 |
| LMRG_01660 | - | propionate CoA-transferase |  |  |
| LMRG_01659 | - | sigma-54-dependent transcriptional regulator | ATTGTGTAATGATGAGCAAA | 1 |
| LMRG_01598 | - | AP-endonuc-2 domain-containing protein | TTTGTGAAAATATTATCAAA | 1 |
| LMRG_01597 | - | NADH oxidase |  |  |
| LMRG_01596 | - | shikimate 5-dehydrogenase |  |  |
| LMRG_01595 | - | MFS domain-containing protein |  |  |
| LMRG_01594 | - | MFS domain-containing protein |  |  |
| LMRG_01978 | cydA | cytochrome bd oxidase subunit I | ATTGTGAAATAAAAAGCAAT | 1 |
| LMRG_01979 | cydB | cytochrome bd oxidase subunit II |  |  |
| LMRG_01980 | cydC | ABC transporter |  |  |
| LMRG_01981 | cydD | ABC transporter |  |  |
| LMRG_02486 | esxA | ESAT-6-like protein | TTTGTGAATTCGTGTTATAA | 2 |
| LMRG_02596 | - | anaerobic ribonucleoside triphosphate reductase | TATGTGAAAGATTTTTCACA | 2 |
| LMRG_02595 | - | highly anaerobic ribonucleotide reductase |  |  |
| LMRG_00154 | - | hypothetical protein | AATTTAAATTGATGGGCAAA | 2 |
| LMRG_00435 | - | hypothetical protein | TTGGTGAAATAATAGCAAAT | 2 |
| LMRG_00476 | - | 2-hydroxyglutaryl-CoA dehydratase | TATGTGAAGCAACGTACAAA  TATGTGAAGTTACATACATA | 2  2 |
| LMRG_00528 | - | myo-inositol-1(or 4)-monophosphatase | TTTGTGATAAAATAAACAAA | 2 |
| LMRG_00858 | pflB | formate acetyltransferase | TTTGAAAAATATTGCACAAA | 2 |
| LMRG_00859 | pflC | pyruvate formate-lyase 1-activating enzyme |  |  |
| LMRG_00865 | - | peptidoglycan bound protein | TTTGTGAAACTTCTTACAAA | 2 |
| LMRG_01453 | glnK | nitrogen regulatory protein P-II | ATTCTGAAATAAATATCAAA | 2 |
| LMRG_01217 | bsh | bile acid hydrolase | TTTGTGAAAAAAACCACAAA | 2 |
| LMRG_01645 | - | hypothetical protein | ATTGTGAAATTTACTAAAAA  TATGTGAACTATTGAATTAT | 2  2 |
| LMRG_02716 | - | FtsX domain-contiaing protein | AAAGTGAAATAGTGTTCATT | 2 |
| LMRG_02717 | - | ABC transporter domain-containing protein |  |  |
| LMRG_01838 | - | hypothetical protein | ATTGTTAATATATTCACAAA | 2 |
| LMRG_02214 | - | hypothetical protein | ATTGTGAAAAAGAAAACAAA | 2 |
| LMRG_02458 | - | DUF4064 domain-containing protein | ATTGCGAAATGTTCATAATT  ATTGTAAACTTCCTTAAAAT | 3 |
|  |  |  |  | 3 |
| LMRG_00004 | - | hypothetical protein | ATTGTGACAATTAGCACAAT | 3 |
| LMRG_00045 | - | long-chain fatty-acid-CoA ligase | TTTGTGATTATATTAACATA | 3 |
| LMRG_00046 | - | fumarate reductase flavoprotein subunit | ATTGTAAAAAAATCTGTAAA  TATGTTAATATAATCACAAA | 3 |
|  |  |  |  | 3 |
| LMRG_00104 | - | hypothetical protein | ATGTTGAATTTCTAGCTAAT  TATGTAATTTTTTTATCATT | 3 |
| LMRG_00126 | inlA | internalin A | TATGTGTTATTTTGAACATA  ATAGTGAAATACATAAAAAT | 3  3 |
| LMRG_00127 | inlB | internalin B |  |  |
| LMRG_00134 | - | hypothetical protein | AATTTCATGTTATATTCAAT | 3 |
| LMRG_00200 | - | lincomycin resistance protein | CTTGTAAATTTTTCGTAAAA | 3 |
| LMRG_00275 | - | formate/nitrite transporter | GTTGTGAATTTCACAAGAAA | 3 |
| LMRG_00332 | - | APA family basic amino acid/polyamine antiporter | TTTTTGTATTTTTTCTCACA | 3 |
| LMRG_02012 | - | formate transporter | TTTGTGATTATTTTAACAAG | 3 |
| LMRG_02114 | - | glycine betaine/proline transport system ATP-binding protein | AATGTGCAAAATGGCGCAAA | 3 |
| LMRG_02116 | - | glycine betaine-binding protein |  |  |
| LMRG_00561 | - | VOC domain-containing protein | TTTTTGATGTTAGAAACAAT | 3 |
| LMRG_00762 | - | hypothetical protein | TTTGTGATAAAATAAACGAA | 3 |
| LMRG_00761 | - | Helicase ATP-binding domain-containing protein |  |  |
| LMRG_00760 | - | PAPS-reduct domain-containing protein |  |  |
| LMRG_00758 | - | ParB domain-containing protein |  |  |
| LMRG_00758 | - | methyltransf-11 domain-containing protein |  |  |
| LMRG_00757 | - | hypothetical protein |  |  |
| LMRG_01332 | lap | bifunctional acetaldehyde-CoA/alcohol dehydrogenase | TTTGTGAAGTTTTTCACGTG  CACGTGAAACACTGGACAAA | 3 |
| LMRG_02521 | - | hypothetical protein | ATAGTTAATTAAAATACAAT | 3 |
| LMRG_02823 | - | gram-pos-anchoring domain-containing protein | AATCTGAAGTGAAGAACCAT | 3 |
| LMRG_02822 | - | hypothetical protein |  |  |
| LMRG_01064 | pflA | formate acetyltransferase | TAAGTGAAAAGGAACACAAT | 3 |
| LMRG_01092 | - | hypothetical protein | TACGTGAAAAAATATACAAA  CTTGTTTATTAATGCACAAA | 3  2 |
| LMRG_01106 | fhuD | iron complex transport system substrate-binding protein | TTTGTGAATAAAGTTGCTAT | 3 |
| LMRG_01105 | - | iron complex transport system permease |  |  |
| LMRG_01104 | - | iron complex transport system permease |  |  |
| LMRG_01267 | - | heme peroxidase | TTAGTGATATTCTAAACAAG | 3 |
| LMRG_01681 | - | hypothetical protein | CTTGCGATATTTTTCACAAA | 3 |
| LMRG_01620 | hemE | uroporphyrinogen decarboxylase | TTTGTTTGGTTTTATACAAA  ATTGTGTGTAAATGAACAAA | 3  3 |
| LMRG_01621 | hemH | ferrochelatase |  |  |
| LMRG_01814 | gadD3 | glutamate decarboxylase | ATTGTGAAAAATTGAACGGT | 3 |
| LMRG_02187 | - | serine/threonine protein phosphatase | TTTTGGAACTTATTGTGAAA | 3 |
| LMRG_02235 | - | magnesium-translocating P-type ATPase | AAAGTTAAGTATTTTACATT | 3 |
| LMRG_02234 | - | cell division protein |  |  |
| LMRG_02233 | - | hypothetical protein |  |  |
| LMRG_02235 | - | DUF5626 domain-containing protein |  |  |

Highlighted genes are predicted to be in an operon [1].

^a^Consensus ROP sites in *L. monocytogenes* were predicted using the *B. subtilis* ROP consensus (5'-WWTGTGAANTNNTNNNCAAW-3') [2].

**Table F.** *L. monocytogenes* strains used in this study.

| **Strain** | **Description** | **Reference or source** |
| --- | --- | --- |
| 10403S | wt | [3] |
| MLR-L752 | *∆rex* | This study |
| MLR-L957 | ∆*rex* p-*rex* | This study |
| MLR-L955 | *∆bsh* | This study |
| MLR-L956 | ∆*rex∆bsh* | This study |
| DP-L4404 | ∆*inlAB* | [4] |
| MLR-L963 | ∆*rex*∆*inlAB* | This study |
| MLR-L1003 | ∆*rex∆inlAB* p-*rex* | This study |

**Table G.** *E. coli* strains used in this study.

| **Strain** | **Description** | **Reference or source** |
| --- | --- | --- |
| XL1 | For vector construction | Stratagene |
| SM10 | For *trans*-conjugation | [5] |
| MLR-E011 | pKSV7-oriT | [6] |
| MLR-E540 | pKSV7x | [7] |
| MLR-E893 | pPLIM1 | Gift from A. Rietsch |
| MLR-E006 | pPL2 | [8] |
| MLR-E964 | SM10/pKSV7∆*rex* | This study |
| MLR-E966 | SM10/pPL2.pNative.*rex* | This study |
| MLR-E965 | SM10/pKSV7x∆*bsh* | This study |
| MLR-E967 | SM10/pLIM1∆*rex* | This study |

**REFERENCES**

1. Wurtzel O, Sesto N, Mellin JR, Karunker I, Edelheit S, Bécavin C, et al. Comparative transcriptomics of pathogenic and non-pathogenic Listeria species. Mol Syst Biol. 2012;8: 583. doi:10.1038/msb.2012.11

2. Ravcheev DA, Li X, Latif H, Zengler K, Leyn SA, Korostelev YD, et al. Transcriptional Regulation of Central Carbon and Energy Metabolism in Bacteria by Redox-Responsive Repressor Rex. Journal of Bacteriology. 2012;194: 1145–1157. doi:10.1128/JB.06412-11

3. Bécavin C, Bouchier C, Lechat P, Archambaud C, Creno S, Gouin E, et al. Comparison of Widely Used Listeria monocytogenes Strains EGD, 10403S, and EGD-e Highlights Genomic Variations Underlying Differences in Pathogenicity. MBio. 2014;5. doi:10.1128/mBio.00969-14

4. Bakardjiev AI, Stacy BA, Fisher SJ, Portnoy DA. Listeriosis in the Pregnant Guinea Pig: a Model of Vertical Transmission. Infection and Immunity. 2004;72: 489–497. doi:10.1128/IAI.72.1.489-497.2004

5. Simon R, Priefer U, Pühler A. A broad host range mobilization system for in vivo genetic engineering: transposon mutagenesis in Gram-negative bacteria. Nat Biotechnol. 1983; 784–791. doi:10.1038/nbt1183-784

6. Camilli A, Tilney LG, Portnoy DA. Dual roles of plcA in Listeria monocytogenes pathogenesis. Mol Microbiol. 1993;8: 143–157.

7. Whiteley AT, Ruhland BR, Edrozo MB, Reniere ML. A Redox-Responsive Transcription Factor Is Critical for Pathogenesis and Aerobic Growth of Listeria monocytogenes. Freitag NE, editor. Infect Immun. 2017;85: e00978-16. doi:10.1128/IAI.00978-16

8. Lauer P, Chow MYN, Loessner MJ, Portnoy DA, Calendar R. Construction, characterization, and use of two Listeria monocytogenes site-specific phage integration vectors. Journal of Bacteriology. 2002;184: 4177–4186.
