# Supplementary material for: The redox-responsive transcriptional regulator Rex represses fermentative metabolism and is required for Listeria monocytogenes pathogenesis
Source: PLoS Pathog. 2021 Aug 16;17(8):e1009379. doi: 10.1371/journal.ppat.1009379 (PMC8389512; doi:10.1371/journal.ppat.1009379)
Supplement: Attachment — Submitted filename: Response to Reviews FINAL.pdf [file ppat.1009379.s007.pdf]

MAJOR ISSUES

Reviewer #1:

1. *Complementation of ∆rex strains. Whereas the authors have generated a complemented ∆rex strain by introducing an ectopic version of rex in the genome, they only used these strains in some of their experiments. This could potentially mask side-effects of the initial deletion, either by a polar effect at the mutated locus, or in case another mutation occurred concomitantly with the deletion in the genome of that strain, on some of the assessed phenotypes. It would thus be important to include the use of complemented strain in:*
   A. The in vivo experiments reported in Fig. 5;

   We repeated the oral listeriosis model of infection and included the *rex* complemented strain (n=30 mice). These data show that expressing *rex in trans* in the pPL2 locus of the ∆*rex* mutant fully complements all observed phenotypes during infection and are now included in **new Figure 5.** We appreciate this comment, as the new results have improved the manuscript.

   B. The experiments which were carried-out to check by RT-qPCR the expression of six Rex targets as a validation of the transcriptome analysis (see also comment #2).

   The validation qPCR was added as **new S1 Fig**, which includes the wt, ∆*rex*, and complemented strain grown in both aerobic and anaerobic growth conditions. More details are explained below.

2. *Validation of the transcriptome. So that the reader can better assess the effect of Rex-mediated repression on its targets, the results of RT-qPCR experiments that were performed to validate the transcriptome should be included as a supplementary figure, together with data from a complemented strain. Moreover, because Rex seems to be exerting its repressive function during aerobic, but not anaerobic growth, it would be important to include both growth conditions in these graphs. Among the genes to be validated, if not those already assessed, it would be important to include the targets that the authors investigated in this work (especially bsh, inlAB, and at least 1or 2 of the metabolic genes). The assessment of rex expression in both aerobic and anaerobic conditions would also be informative, as well as expression in acid and neutral bile, aerobic and anaerobic conditions for rex and bsh (see comment #3).*

   We validated the transcriptome analysis by qPCR and included the complemented strain grown in aerobic and anaerobic conditions (**new S1 Fig**). As expected, following 7 hours of aerobic growth in BHI, transcript levels of 5 representative genes were increased in the ∆*rex* mutant compared to the wt and *rex* complement strains (new S1A Fig). We also validated that Rex repression was alleviated during anaerobic growth by assessing the transcript levels of the same 5 genes from RNA isolated from anaerobically grown bacteria at stationary phase (new S1B Fig). Here, transcript levels were similar between the wt, ∆*rex,* and the complemented strains with the exception of lap transcript, which was increased 10-fold in ∆*rex* at 7 hours post-inoculation (new S1B Fig). Increased lap transcripts in the ∆*rex* mutant was not unexpected, as this strain produces more ethanol than wt at 6 hours post-inoculation during anaerobic growth (**new S2H Figure**). These results suggest that while Rex repression is alleviated during log growth under anaerobic conditions, Rex repression is exerted following glucose depletion and entry into stationary growth **(new S2G Figure).**

   We also compared rex transcript levels in wt and the complemented strains under both aerobic and anaerobic conditions. Under anaerobic conditions, we observed a decrease in *rex* transcription (**Figure below**). This is consistent with what has been reported in *S. aureus,* in which Rex does not regulate itself (Pagels 2010)*.* Indeed, our *in silico* analysis of the *L. monocytogenes* genome did not predict a Rex binding site within the promoter sequence of *L. monocytogenes rex,* suggesting that Rex does not regulate itself*.* Because we have no experimental data describing how *rex* expression is regulated in *L. monocytogenes,* we do not think these data add to the overall conclusions of the manuscript.

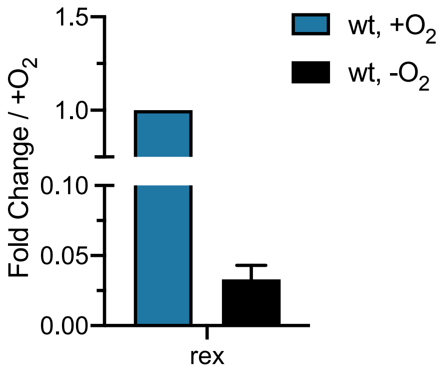

While it was an excellent idea, we were unable to assess bsh transcript level in the presence of neutral and acidic bile. In our experience, isolating quality RNA from 24-hour cultures has proven technically difficult. Additionally, bile is very challenging to work with, as it is viscous and we are unable to separate the bacteria from the bile via centrifugation or filtration. However, the *L. monocytogenes* response to acidic bile has previously been published and they found that exposure to pH 5.5 induces the PrfA and SigB regulons, which include *bsh* (Guariglia-Oropeza 2018). We added a description of this finding to lines 366-368.

Among the targets to be validated in the mutant and complemented strains for assessment of potential polar effects, beyond bsh (LMRG_01217) which is located within 5,000 base pairs of rex in the genome, LMRG_01221 and LMRG_01222, which are located immediately nearby rex and induced in ∆rex in stationary phase would also deserve testing.

Polar effects are highly unlikely due to a number of factors. First, transcript levels of LMRG_01218 and LMRG_01219, which are located directly downstream of *bsh* (LMRG_01217), were not changed in the transcriptome analysis. Second, LMRG_01221 was altered in expression in our transcriptomics, but it is encoded on the opposite strand, making polar effects especially unlikely. Finally, there are multiple transcription terminators between LMRG_01221/22 and *rex*. However, to directly measure potential polar effects of a *rex* deletion, we measured transcription of LMRG_01221 (*lmo2070*), which is the first gene in the operon directly upstream of *rex*. As expected, we observed an increase in LMRG_01221 transcript in the ∆*rex* mutant that was restored back to wt by expressing *rex* using pPL2 (∆*rex* + *rex*). These results indicate that increased transcription of LMRG_01221 in ∆*rex* is not due to polar effects because the pPL2 locus is over 492,000 bp away from the *rex* chromosomal site. We have included those data here; since we did not mention this operon in the text, we thought it would be confusing to add to the manuscript. However, we will happily add it if you and/or the editor feel it is integral to our conclusions.

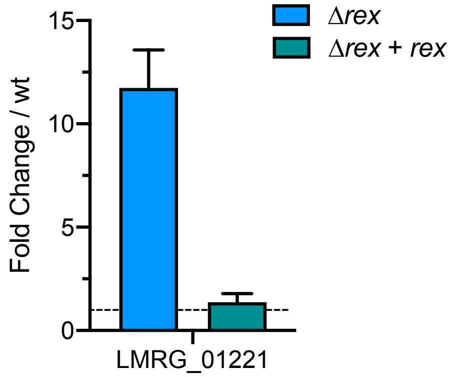

3. *Fig. 2. Role of Rex in bsh regulation and resistance to acid bile in anaerobic conditions.*
During anaerobic growth in acid bile, the main difference that is seen with the ∆rex mutant strains came as non-significant in statistical testing. When looking at the data, it seems to proceed from a single data point behaving differently to the rest, which might as well be an experimental outlier. This would match the author's statement that "Rex is typically derepressed during anaerobic growth", hence wt and ∆rex are not expected to differ in this condition. In addition, the interpretation provided in the text (P11 L188-200) when

*discussing the role of Rex on Bsh in the phenotype of the ∆rex∆bsh mutant is unconvincing, because in fact no effect of Bsh on survival in acid bile in anaerobic conditions is seen in this set of data, when comparing at the wt and ∆bsh strains. As stated by the authors, Rex is not active in anaerobic conditions; therefore in the wt strain bsh is expected to be expressed (unless it is repressed by something else in a Rex-independent way). In case Bsh was playing a role in acid bile resistance in this condition, viability of the ∆bsh strain should be lower than that of the wt strain (as was the case in aerobic conditions), which does not seem to be the case. Then, the authors attribute, in the ∆rex strain, the increase in resistance to an increase in bsh expression, although (i) they did not assess this expression increase compared to the wt in anaerobic acid bile (see comment #2) and (ii) they assume that, due to the fact that Rex is not active in anaerobic growth, this increase in expression in the ∆rex mutant compared to the wt is "likely the result of activation by unknown factors" (i.e. Rex-independent, contradicting the statement L194 that "the increase in survival of ∆rex was dependent on bsh"). Because the effect of ∆rex is not significant, because phenotypes are unconvincing, and because gene expressions underlying these phenotypes were not assessed**, I suggest removing Fig. 2B and L189-197 from the results unless this experiment is consolidated.** This would not be of any damage to the overall message of the article, since Rex is not expected to play a role in anaeroby. The last sentence of the paragraph would then read "Taken together, these results demonstrated that L. monocytogenes lacking the Rex repressor are more resistant to acidified bile due to increased bsh expression during aerobic growth".*

In addition to this comment, the two other reviewers expressed concerns about Fig 2B. As you have suggested, we deleted Fig 2B for clarity. Indeed, transcription of *bsh* was not increased in the ∆*rex* mutant during anaerobic growth (new Fig S1B) and therefore, we would not expect any increased survival in this strain. Survival in acidified bile under anaerobic conditions was tested multiple times (n=5) with varied results in all strains tested. This is actually fairly common for bile assays; since bile is a complex biofluid, there is often batch-to-batch variation. We hypothesize that under these conditions, a lower percentage of bile could produce more consistent results for all strains. However, we do not believe these experiments would contribute to the overall conclusions of this work and have decided to take your suggestion and remove Fig 2B.

<u>Reviewer #2:</u>

1. *The authors use in silico analysis to suggest that they have identified Rex binding sites in Listeria. Direct demonstration of binding to a representative site would affirm the notion that rex is a direct repressor.*

   While we agree that direct binding of Rex to the various promoter sites would be valuable, these experiments are not trivial. For example, entire manuscripts have been devoted to investigating Rex proteins from *B. subtilis* and *S. aureus* and evaluating binding affinity to various promoters in the presence and absence of NADH, NAD+, and ATP (Wang 2008 and Pagels 2010). Because the *L. monocytogenes* Rex predicted DNA binding site is so similar to that of the biochemically characterized homologues, we are fairly confident it is directly binding these sites. However, in light of your concern, we changed the wording in the manuscript to eliminate the claim of "direct" Rex regulation, which does not change our overall conclusions.

2. *Line 129 mentions genes "activated" by Rex. This does not seem to be an accurate statement as it implies Rex is a direct activator, which the authors assert is not likely to be true. There may be a better choice of words here.*

   Thank you for pointing out this confusion. We changed our wording here to "genes activated in the presence of Rex," (new L127-28)

3. *Line 191-195 and Fig 2B. The statement that a rex mutant displayed a 20-fold increase in survival compared to WT is based off of a dataset where one data point biases the outcome, so it is difficult to make a conclusion one way or another. The possibility that a rex mutant cannot survive in 0.1% bile at pH5.5 when grown anaerobically is an equally viable conclusion, given the data. The experiment would have to be repeated with an increased number of replicates to make a conclusion on the increased survival of the rex mutant in this specific condition and the role of bsh in mediating that survival.*

As the two other reviewers also expressed concerns about Fig 2B and even suggested deleting it, we decided to remove Fig 2B for clarity. Indeed, transcription of *bsh* was not increased in the Δ*rex* mutant during anaerobic growth (new Fig S1B) and therefore, we would not expect any increased survival in this strain. Survival in acidified bile under anaerobic conditions was tested multiple times (n=5) with varied results in all strains tested. This is actually fairly common for bile assays; since bile is a complex biofluid, there is often batch-to-batch variation. We hypothesize that under these conditions, a lower percentage of bile could produce more consistent results for all strains. However, we do not believe these experiments would contribute to the overall conclusions of this work and have decided to take the suggestion of Reviewer #1 and remove Fig 2B.

4. *The only case where invasion appears to be impacted by a rex mutant is caco-2 cells. All other cell types (Huh-7, TIB73, and BMM) do not show any discernable differences at the 2-hour timepoint or at any timepoint thereafter (a consequence of increased invasion). Thus, it is unclear at this point whether or not the increased gene expression of inlA and inlB mediated by Rex manifests in any measurable way, or at all, during infection. The connection is not felt to be sufficiently justified based on the experimental evidence provided.*

These tissue culture invasion assays were meant to validate the changes we observed by RNAseq, in that increased *inlAB* expression should lead to increased invasion. We repeated the invasion assays to include the complemented strain (in response to Reviewer 3) and now report increased invasion of Δ*rex* into Caco-2 and Huh7 cells, as compared to wt. These data therefore validate the RNAseq results. However, these in vitro phenotypes are relatively small, and therefore unlikely to be evident during infection of a whole animal. Further, bacterial uptake into BMMs (and other phagocytes) is independent of InlAB and therefore we would not expect changes in Δ*rex* invasion into BMMs. We added several sentences describing these results more thoroughly (L316-320).

5. *The dramatic decline in CFU in the gall bladder despite initially productive infection at day 1 is very cool, but is not congruent with the survival data for a rexA mutant in porcine bile. The animal data suggests that rex-mediated repression is quite important in the gall bladder despite its control over bsh. This makes it challenging to relate the in vitro findings to the in vivo condition. This is discussed by pointing out major unknowns about the gall bladder environment. Is there any way to more concretely reconcile these differences? Perhaps based on other gene expression changes that occurred?*

We agree that it is challenging to relate the *in vitro* bile survival findings to the *in vivo* gallbladder data, which is what makes us excited about these *in vivo* results. The only thing known about the contents of the gallbladder is the composition of bile. However, bile at neutral pH (as it is within the gallbladder) is not toxic to *L. monocytogenes* (Fig 2). Therefore, we do not hypothesize that increased transcription of *bsh* in the Δ*rex* mutant has any effect on bacterial survival in the presence of neutral bile in the gallbladder. While it has been demonstrated that *L. monocytogenes* strains lacking *bsh* are required for virulence within the GI tract during infection (Begley 2005, Dussurget 2002), nothing is known about the requirement of *bsh* in the gallbladder during infection. In fact, the researchers investigating the function of *bsh* did not examine the gallbladder. We now explain this more thoroughly in lines 345-358.

6. *Lines 278-280. It is stated that rex-dependent regulation is essential for surviving and colonizing the gall bladder. This statement is at odds with the data. The bacteria seem to get to the gall bladder just fine (infect?), and in some cases (not all) they either die off or are eliminated from the gall bladder by extrusion. Whatever the ultimate mechanism, two of the five mice still had a substantial number of CFU in the gall bladder, so the use of the term essential does not seem to be an accurate reflection of the data.*

You are correct in stating that Δ*rex* bacteria do initially colonize the gallbladder so we have re-worded this statement to reflect that while the bacteria are able to reach the gallbladder initially, they do not survive within this organ over time (now line 264-265). However, compiling the data from all 3 independent infection studies, we were only able to recover significant numbers of Δ*rex* CFUs from 2 of the 13 gallbladders of mice on day 4 of infection (**Fig 5H, S4H Fig, and S5F Fig**), suggesting that Rex regulation is required for full virulence in this organ.

7. Lines 286-287. What is the evidence that Rex directly regulates virulence factor production during infection? The in vitro broth culture data suggest that it might occur, but there was no data to directly support regulation of virulence factors by Rex in vivo.

We reworded this statement: “We also present evidence that the presence of Rex impacts virulence factor production *in vitro.*” (now lines 271-272)

8. Lines 310-311 Is acetate fermentation really an "end-product" of aerobic growth? Acetate typically accumulates during overflow metabolism when there is excess carbon source available. The fact that it accumulates during aerobic growth is probably more a by- product of excess glucose in the medium, rather than it being an end-product of aerobic metabolism per se. Perhaps modify the language used?

We modified the language used here (now line 293).

Reviewer #3:

1. According to the literature, the regulation of Rex goes along with other stress regulators such as sigma B , and hence its examination under BHI conditions is probably masking for its true role. Personally, I think that these are not the best conditions to test the role of Rex (this is just a general comment, you don’t have to do with it anything).

Thank you for this comment and we agree that the use of rich media could be masking more prominent phenotypes in *in vitro* studies of the Δ*rex* mutant. We now comment on this in line 295. We also made a comment in the discussion about the overlapping regulators (now lines 364-373).

2. *It is necessary to validate the RNA seq data by RT-qPCR, at least for the genes that are investigated in the manuscript. The RT experiments should be done in aerobic and anaerobic conditions in WT versus delta-rex. It should be shown that the rex-dependent genes are indeed induced upon anaerobic growth in the presence of Rex, and even inhibited by Rex when over-expressed under this condition. These experiments will help to connect the identified genes with Rex-regulation under aerobic and anaerobic conditions.*

We have included the validation of the RNA-Seq data by qPCR as **new S1 Fig**, in both aerobic and anaerobic conditions. As expected, the 5 genes measured were increased in the Δ*rex* mutant during aerobic growth and transcripts could be restored to wt levels when complemented *in trans* (new S1A Fig). Furthermore, transcript levels were unchanged in the Δ*rex* strain compared to wt when grown anaerobically, indicating that Rex repression is alleviated under these conditions (new S1B Fig).

We appreciate the suggestion to over-express Rex to potentially exert more repression in the wt strain. However, during aerobic growth over-expression of *rex* in wt *L. monocytogenes* utilizing a pPL2.pHyper over-expression construct (*rex*OE) only increased repression of 1 of the 4 representative genes tested from the RNA-Seq analysis (**Figure below**). As we do not yet understand the regulation of Rex itself and how Rex protein levels effect downstream regulation of target genes, we cannot clearly interpret the results from *rex*OE strain qPCR experiments. Importantly, abundance of Rex protein is not expected to affect Rex binding to DNA, as the NADH and NAD+ concentrations are unaltered. Future studies aim to address these questions.

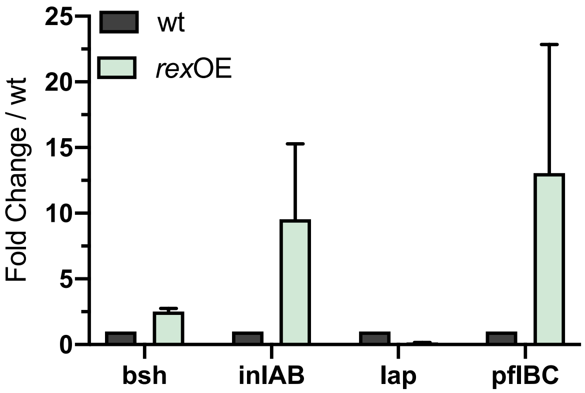

3. *Line 138-141: Figure 1B, the growth defect of delta-rex is minor and might be due to the high expression of its regulated genes, e.g., lap, which is highly induced (342-fold). The conclusion that Rex is dispensable under anaerobic growth is not supported by the presented data, as according to the growth curves, it seems to be dispensable also under aerobic conditions. In this regard, it is strange that the authors could not detect ethanol production. What method was used?*

   We have changed the wording to better reflect the data: "In contrast, the Δ*rex* strain exhibited no growth defect when incubated anaerobically (**S2A Fig**), demonstrating that Rex-dependent repression is alleviated during anaerobic growth" (now lines 137-139).

   Extracellular metabolites were measured in the supernatants of bacterial cultures using the Roche Yellow Line Kits from R-Biopharm. We were able to detect ethanol production during anaerobic growth, when cultures were sealed within an anaerobic tube (**S2B Fig**). However, we were unable to detect ethanol in aerobic cultures because it is a volatile substance and evaporates when shaking in a flask at 220 rpm (L148-156).

4. *Figure 1 D-G; a control is missing. The concentrations of these metabolites should be shown in WT bacteria grown in aerobic versus anaerobic conditions, to demonstrate the switch to fermentative metabolism, as well as in comparison to delta-rex, to examine its role in this regulatory switch.*

   As you suggested, we **revised Fig 1** to include the anaerobic metabolite concentrations side-by-side with the aerobic metabolite concentrations. Presenting the data in this way demonstrates an increase in lactate (Fig 1D) and formate (Fig 1E) production in wt when grown anaerobically, indicating a switch to fermentative metabolism. We thank the reviewer for this suggestion, as it better clarifies the metabolic data.

5. *Also in Fig 1C, a positive control for changes in glucose consumption is needed.*

   We are not sure what a positive control for glucose consumption would be. This experiment measures the amount of glucose in the supernatant of cultures over time, so wt serves as the control for normal *L. monocytogenes* glucose consumption in these growth conditions. The Δ*rex* mutant consumes glucose from the media at the same rate as wt, therefore we conclude that the slight growth defect seen in aerobically grown Δ*rex* is not a result of decreased glucose consumption.

6. *Fig 2A: the transcription level of bsh gene should be analyzed under the tested conditions, i.e., 0.1% bile and 0.1% bile+pH5.5 in WT and delta-rex mutants, to better connect the regulation of Bsh to the phenotypes.*

   While it was an excellent idea, we were unable to assess bsh transcript level in the presence of neutral and acidic bile. See comment above for more information (Reviewer 1, comment 2).

7. *Lines 192-197 (Fig 2B) should be rephrased, the phenotype of delta-rex is insignificant, and hence any interpretation of the data is not contributing.*

As the two other reviewers also expressed concerns about Fig 2B and even suggested deleting it, we decided to remove Fig 2B for clarity. See above for more information (Reviewer 1, comment 3).

8. *It is worth over expressing Rex in WT bacteria grown under aerobic and anaerobic conditions with bile to see if the survival of the bacteria reduces.*

As mentioned above in comment 2, over-expression of *rex* does not result in more repression of target genes, including *bsh.* As we do not yet understand the regulation of Rex itself and how Rex protein levels affect transcription of its target genes, these experiments would not be interpretable.

9. *Line 186-7, The data of the double mutant indicates that bsh deletion has a dominant effect. It will be nice to see if WT bacteria become resistant if over-expressing bsh (from pPL2).*

This was an excellent idea that we spent a great deal of effort on; however, we were ultimately unsuccessful in generating an over-expressing strain of *bsh.* Over-expression of *bsh* using a pPL2.pHyper construct was toxic to our recipient strains of *E. coli,* resulting in major *bsh* gene rearrangements following transformation.

10. *Line 196- rex/bsh transcription during anaerobic growth should be measured by RT-PCR.*

While we deleted this line from the text, qPCR demonstrated that *bsh* transcription is not increased in Δ*rex* compared to wt under anaerobic conditions (new **S1B Fig**).

11. *Fig 3 A-D. What are the p values for the differences between Δrex and Δrex pPL2rex? they seem to be not statistically significant. This could be problematic. Moreover, the phenotypes of the double mutant (with InlAB) could be due to the dominant effect of the internalins.*

It has been published by many groups that strains lacking inlAB are completely deficient in host cell invasion in cell culture, so we predicted the effect of the internalins would be dominant. Moreover, these tissue culture invasion assays were meant to validate the changes we observed by RNAseq, in that increased *inlAB* expression should lead to increased invasion. We repeated the invasion assays to include the complemented strain and now report increased invasion of Δ*rex* into Caco-2 and Huh7 cells, as compared to wt. In Caco-2 cells, the *p* value between Δ*rex* and Δ*rex* p-*rex* was 0.0143, while in Huh7 cells it was 0.02.

12. *229-231, the conclusion here is wrong. The data says that the rex-regulated genes are not required for intracellular growth, and not that the rex regulated genes are de-repressed during intracellular growth, that was not tested.*

This conclusion was reworded. It now reads: "These results suggested that Rex-mediated repression is not required for intracellular growth and therefore, deleting the Rex repressor had no effect on growth." (now L217-219)

13. *Can the authors show the intracellular growth curve in Caco-2, is there any defect?*

The intracellular growth curve of wt and Δ*rex* in Caco-2 cells is shown in the figure below (MOI of 10, n=2). While there is a slight decrease in Δ*rex* CFU at 6 hours post-infection, this change was not significant. We did not include this growth curve in the final manuscript, as the Δ*rex* mutant did not have a defect within the GI tract of mice so this cell type is not relevant. Because there was significant attenuation in the livers of mice infected with Δ*rex*, we decided it was more informative to include growth curves in activated macrophages and both human and mouse hepatocyte cell lines (Fig 4A-C).

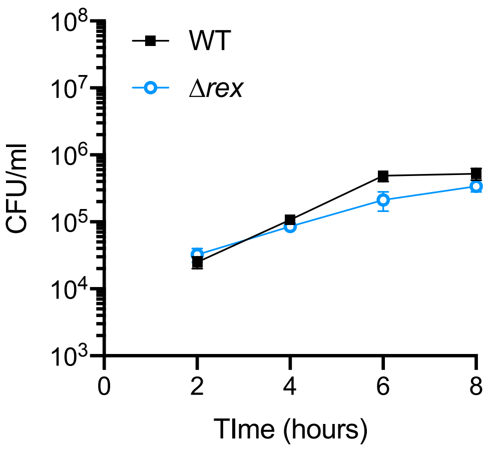

14. Line 275-6. Rex repression can be tested experimentally. This is a major point in the model suggested by the authors, yet it is based on speculation and not on experimental data. In addition, ectopic expression of Rex may be helpful to decipher its importance in the in-vivo infection model.

Over-expression of Rex in wt *L. monocytogenes* is not sufficient to repress target genes in the absence of NADH:NAD+ manipulation (see comment #2 above) so ectopic expression *in vivo* is unlikely to be informative. We reworded the text to say "Taken together, these results confirmed our hypothesis that Rex-dependent repression is not required for colonization and invasion of the GI tract during oral infection as a Δ*rex* mutant was able to colonize and disseminate from the GI tract similar to wt. These results suggest that Rex is normally derepressed in this environment" (now lines 260-263).

15. Figure 5: this is the most important figure of the manuscript; however several issues arise:
It is stated in the legend that solid line indicates the median of 5 mice per group. This representation is meaningless for n=5 and highly misleading. These should be presented as mean and error bars for standard deviation.

Thank you for bringing this to our attention. We agree that using the median for these data can be misleading, as it can exclude extreme outliers. However, because we do have variability in these data with outliers, using the arithmetic mean would skew the data towards these outliers. Therefore, we now present the geometric mean, which takes outliers into account but is not too sensitive to them (Martinez 2017, Pharmaceutics). We changed Figure 5 (and the corresponding S4 Fig and S5 Fig) to report the geometric mean instead of the median.

I guess that panel A shows mean and stdev, but it is not stated in the legend.

We corrected the legend to reflect the data reported.

The authors should add the second biological repeat as a supplementary figure to give an estimation of the biological reproducibility.

We have now included all 3 replicates of the *in vivo* data in the manuscript (**Fig 5, S4 Fig, and S5 Fig**).

The experiments should include the complemented strain Δrex-pPL2-rex.

The complemented strain Δ*rex* p-*rex* has been included in Fig 5.

Fig 5B shows a 3-log difference at day 1, while the other panels do not. Can the authors comment on the biological relevance of that, or that this is a technical problem?

Some variability is expected in the oral model of infection (Bou Ghanem 2012 and Pitts 2018). By 24 hours post-infection, bacteria have colonized the gallbladder and thus, are being expelled along with the bile back into the GI tract following the ingestion of food. We hypothesize the variability seen in the small

intestinal tissue could be due to differences in the frequency of eating between individual mice and frequency of bacteria being expelled by the gallbladder. However, this mechanism has not been experimentally tested.

Since the authors suggest that Rex is not required in the GI, similar results for dissemination to organs are expected also upon IV infection. Did the authors perform these experiments?

Following the construction of the Δ*rex* strain we initially performed a 48-hour intravenous infection and found no difference in bacterial burdens between wt and Δ*rex* in the spleen or liver (Figure below). This is consistent with results from our oral infection in which Δ*rex* is able to disseminate to these organs similarly to wt. As this was an initial pilot experiment, we do not have sufficient numbers to publish these data in the manuscript.

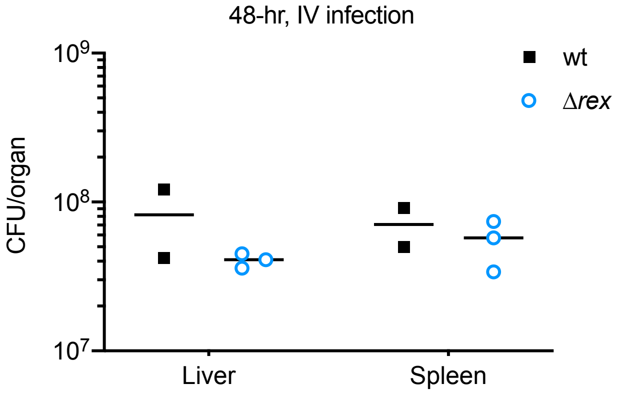

Lastly, the suggested role of Rex is not in line with the in vivo data (this mutant is attenuated in mice). As well as the bile data is not in accordance with the survival of the bacteria in the gallbladder. Can the authors comment on that?

We appreciate this comment from the reviewer, as it reveals that we did not clearly explain our overall conclusions in the previous version. In lines 345-358, we point out that while other mutants have been found to be defective in the gallbladder, these mutants were either lacking known virulence factors or sensitive to bile stress *in vitro.* In contrast, the Δ*rex* strain is insensitive to neutral bile, more resistant to acidified bile stress, and is not impaired for intracellular growth or intercellular spread. We believe this highlights the novelty of the work presented in this manuscript as it demonstrates that (i) *in vitro* phenotypes do not always correspond with *in vivo* findings and (ii) that bile is not the only stress encountered during gallbladder colonization. We are excited to continue our investigation aimed at identifying other stressors present in the gallbladder and the mechanisms used by *L. monocytogenes* to survive in this environment.

MINOR ISSUES

Reviewer #1:

4. *Fig. 3. Role of Rex in inlAB regulation and effect in cell adherence/internalization. According to the description in the methods section (p. 23), the ΔrexΔinlAB strain that was used in Fig. 3 was not obtained by deleting inlAB in the Δrex strain that had been previously generated, and that was checked by pPL2rex complementation on the same graph, but by deleting rex in a ΔinlAB strain previously generated in another lab. In addition, another vector (pLIMΔrex) was used for generating this construct than the pKSV7Δrex construct used when generating the Δrex strain. Altogether, this makes it difficult to assess whether the ΔrexΔinlAB strain might not suffer from other mutations introduced during rex mutagenesis than rex deletion itself.*

We appreciate this comment and the thorough examination of our methods. There were technical difficulties using pKSV7Δ*rex* to delete *rex* in the Δ*inlAB* mutant during passaging to cure the integrated plasmid. Therefore, we utilized the pLIM construct, which does not require passaging and instead uses

counterselection with *p*-chloro-phenylalanine. To address your concern, we complemented the Δ*rex*Δ*inlAB* mutant with pPL2.*rex* which was tested in the invasion assay. As expected, complementation of *rex* had no effect on invasion when *inlAB* was absent in either Caco-2 epithelial cells or Huh7 hepatocytes (revised **Fig 3**).

5. *Fig. 5. Phenotype in the gallbladder. In the abstract, the authors make a point that the Δrex mutant was "nearly sterilized in the gallbladder". This notion of a phenotype that would be more pronounced in the gallbladder than in other deeper organs analyzed (spleen, liver) is also emphasized in the discussion section, although not strongly supported by the data provided, given (i) the high variability in counts in this organ and (ii) the fact that the mutant was not complemented (see comment #1). This insistence on a phenotype in the gallbladder may also bring confusion to the reader, due to the repressive role of rex on bsh and the effect of the Δrex mutant in acid bile, by a juxtaposition effect when flipping through the abstract without background. In the current state of the data, being less affirmative about a role in the gallbladder might be cautious. Related with that, P19 L365, "specifically" may be too strong, as the authors do not really show that Δrex is more "specifically" affected in the gallbladder than elsewhere. Lower counts on average plus high variability in this organ could also proceed from bottleneck effects on the small counts of bacteria being liberated from the liver.*

We believe the emphasis on the gallbladder phenotype is valid for a few reasons:
1) After compiling the data from all 3 independent infection studies, we found that only 2 of the 13 mice infected with Δ*rex* harbored more than 10 CFU in the gallbladder 4 days post-infection. This was in stark contrast to mice infected with the wt or complemented strain, which each harbored 10^6-10^7 CFU per gallbladder at this time point. We now include all these data as Fig 5H, S4H Fig, and S5F Fig.
2) The Δ*rex* p-*rex* strain fully complemented *in vivo* with very minor variability at day 4 (**new Fig 5H**).
3) While a handful of other studies have described mutants defective in gallbladder colonization, they were all either lacking known virulence factors or sensitive to bile stress *in vitro.* In contrast, the Δ*rex* strain is insensitive to neutral bile, more resistant to acidified bile stress, and is not impaired for intracellular growth or intercellular spread. Together, these data suggest that the dramatic attenuation of Δ*rex* in the gallbladder is due to other factors and not simply bile stress, as has been previously assumed. We explain these results more thoroughly now in lines 345-358.
"Specifically" was removed from L365 (now L356).

6. Mouse model. The authors used a conventional mouse model, rendered more susceptible to listeriosis thanks to antibiotic treatment prior to gavage. However, conventional mice are non-permissive to InlA-mediated entry in epithelial cells, due to an amino-acid change in the E-cadherin receptor (Lecuit et al. EMBO J 1999). Because the authors show that Rex is a regulator of inlA expression, they should at least mention in the discussion that, in case Rex regulated InlA-mediated invasion in vivo, the chosen mouse model would have been blind to any possible consequence on infection. Especially, possible effects on the ability to cross the intestinal barrier or to colonize tissues may have been missed.

We added a discussion of the InlAB species-specificity to lines 316-320.

7. *It would be informative to indicate the position of rex in the genome (LMRG_01223), for instance in the methods section, in the paragraph dedicated to the description of how mutant strains were generated.*

Thank you for bringing this to our attention. The position of *rex* in the genome has been added to the methods section (now line 411).

8. *Use of "derepression".*
Throughout the text, the used or "Rex derepression" by the authors may be confusing to the reader. Indeed, while the authors mean that Rex target genes are derepressed (that the function of the repressor is alleviated), the reader might interpret this ambiguous wording as the alleviation of a repression that was exerted on rex function (meaning that the repressor would become active). Here follow a few suggestions of rewording to avoid this ambiguity:
P10 L160-1. "demonstrating that Rex-mediated repression is normally alleviated in this growth environment"

now lines 166-167

P11 L196. "As Rex repressor activity is typically turned off during anaerobic growth"

this was deleted from manuscript

P13 L229. "These results suggest that Rex repressive activity on its genes is alleviated in wt L. monocytogenes during intracellular growth"

now lines 217-219

P13 L243. "Rex-mediated repression"

now line 230

P13 L255. "Derepression of Rex targets would not"

now line 232

P16 L285. "that derepression of Rex targets"

now line 269

P17 L318. "when Rex-mediated repression is relieved"

now line 301

P17 L323-4. "Alleviation of Rex-mediated repression increased expression"

now line 305

P18 L 335. "showed that alleviation of Rex-mediated repression coordinates"

now line 321

As suggested by the reviewer, we changed the text to avoid any confusion about the phrase "Rex derepresssion" to the reader.

9. *P19 L356, the statement "although bile was not toxic to L. monocytogenes at neutral pH" is misleading in this position in the text, because it seems to imply that Rex could have been expected to participate in regulating the detoxification against bile (actually, to help resist against bile, since here ∆rex is attenuated). However, the authors showed previously that Rex did not participate in resistance against neutral bile in vitro (Fig. 2A). It is thus useless, and even confusing to readers to relate again Rex to bile only because the gallbladder is mentioned, rather than stating, as for the liver or spleen, that Rex is required for proliferation and maintenance in this organ.*

We deleted this statement from the manuscript.

10. Statistics. In most graphs, statistical testing was performed by heteroscedastic Student's t-test. Because the data tested are in most figures not from two independent groups, but from three or more (for instance in Fig. 2, 5 conditions are tested and compared with each other), one-way ANOVA followed by appropriate post-hoc test would be better suited.

In each experiment that a heteroscedastic Student's t-tests was performed, only two independent groups were being compared to each other. For example, in Fig 2 the asterisks and lines are only denoting significance between the bars each line is pointing to. In other words, wt compared to ∆*rex* or ∆*rex* compared to the complemented strain.

Reviewer #2: (No Response)

Reviewer #3:

Comments about the bioinformatic data:

Line 120-121:"We identified potential Rex binding sites in the promoter regions of 48 genes and/or operons repressed by Rex (Table S5)." How this analysis was conducted? What was the genome analyzed? 10403S? If so the gene tags should be LMRG_XXXX and not LMO.

We added more text in the manuscript detailing how this analysis was done: "*In silico* promoter analysis of genes in the 10403S genome exhibiting Rex-dependent regulation was performed to determine potential Rex binding sites using the *Bacillus subtilis* Rex consensus sequence. Allowing up to 3-

mismatches with the *B. subtilis* consensus sequence, we identified potential Rex binding sites in the promoter regions of 48 genes and/or operons repressed by Rex (S5 Table)." (now lines 116-118)

We did analyze the 10403S genome and have changed the gene tags in Table S5 to reflect this.

Tables S1 and S2, column titles (LMRG and Lmo) should be replaced with names of corresponding strains - 10403 and EGD-e.

Thank you for bringing this to our attention; these changes were made in Tables S1-S4.

A comprehensive info of the Supplementary Tables (S1, S2, and S5) should be updated in terms of 'annotation' of function(s) on many "hypothetical proteins" shown there. The current NCBI and UniProtKB resources can help to do that. For example the following "hypothetical proteins" have an annotation: LMRG_02136 is CRISPR/Cas system-associated protein Cas2 (cl11442); lmo2234 is predicted to be a sugar phosphate isomerase/epimerase (COG1082) and it has an excellent resolution 3D structure (1.7A; PDB: 2G0W); lmo2237, is a putative member of the well-known Major Facilitator Superfamily (MFS) protein (cl28910) etc. This information will give more insight into the impact of Rex.

We appreciate the reviewer's in-depth analysis of the supplemental tables and suggesting a more thorough examination of the hypothetical proteins. We utilized the UniProt database to further analyze the RNA-Seq tables and have re-annotated these lists to reflect this analysis.

The lap gene (LMRG_01332, lmo1634) found in this study as Rex-dependent (342-fold; Tables 1 and S1) is probably one of most abundant in expression even in WT bacteria. Wurtzel et al (2012) showed its hyperexpression in str. EGD-e grown in BHI (see data in their Table S1). Also, the two predicted 3-mismatch Rex ROPs of lap (CACGTGAAACACTGGACAAA, TTTGTGAAGTTTTTCACGTG) should be replaced to be shown according to their chromosome positions (TTTGTGAAGTTTTTCACGTG, CACGTGAAACACTGGACAAA); these two candidates overlap one another – any comments on that!? See a similar story of double and/or overlapping binding sites in CodY regulons of both L. monocytogenes and B. subtilis. Other 'double' ROPs in Table S1 should be verified e.g. of lmo1945.

We have reorganized Table S5 to better reflect the chromosome position of the ROP sites in any promoters with multiple predicted ROP sites. In *B. subtilis*, several Rex-regulated genes contain multiple ROP sites within their promoter regions (*cyd* contains 3 ROP sites, *ldh* contains 2 ROP sites), and Rex binds to each with varying affinities (Wang 2008, Mol Micro). We hypothesize that a similar situation occurs in *L. monocytogenes* where Rex binds to multiple ROP sites within promoters with differing affinities. Further investigation of the functional properties of *L. monocytogenes* Rex are needed to address this hypothesis.

The data presented in Table S5 should be used to get a preliminary consensus of Rex by using WebLogo options (http://weblogo.berkeley.edu/logo.cgi). I used the whole set of 0-3 mismatch predicted ROPs of Table S5 and found that there are some differences between the L. monocytogenes Rex ROP consensus and those of B. subtilis and S. aureus. There is a trend to have W-rich (A or T nucleotides) linker between left and right arms of the L. monocytogenes ROP consensus. Prediction of the 'listerial' consensus of Rex ROP could make the analysis of putative members of the regulon more intriguing.

We appreciate the reviewer's interest in the *in silico* ROP analysis, in which we allowed up to 3 mismatches to be consistent with what has been previously done in the literature (Pagels 2010). Here, we created WebLogos for predicted Rop sequences in *L. monocytogenes* Rex-dependent promoters with 3, 2, or 1 mismatch compared to the *B. subtilis* ROP sequence (A-C below). However, we are hesitant to include WebLogo analysis in this manuscript without experimental data to demonstrate direct binding of Rex to these predicted ROP sites. While we are interested in investigating the DNA-binding properties of *L. monocytogenes* Rex, those experiments are outside of the scope of this manuscript.

A. Up to 3 mismatches

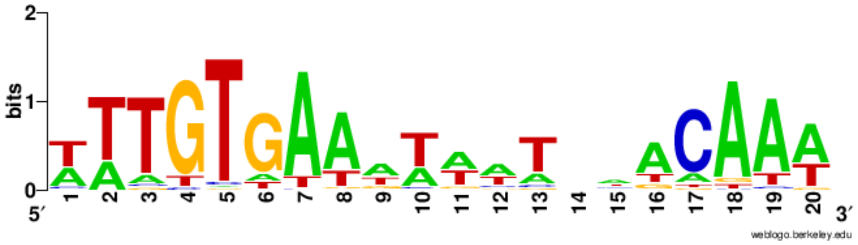

B. Up to 2 mismatches

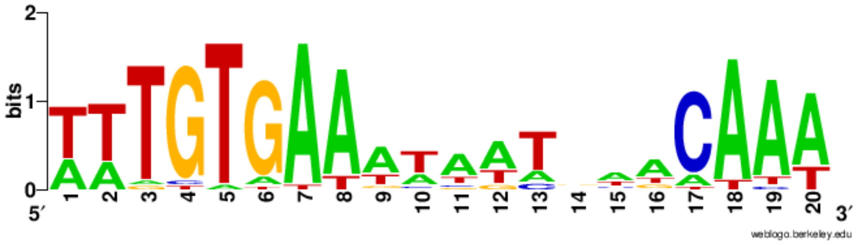

C. Up to 1 mismatch

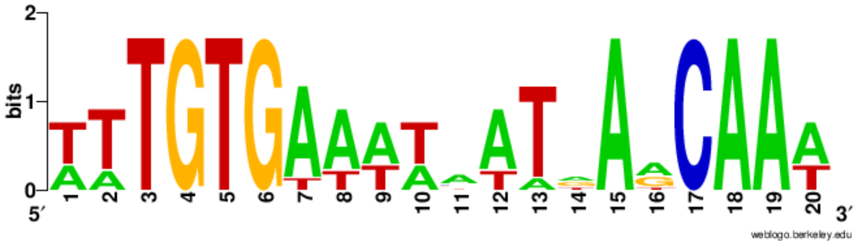

Hecker and his colleagues wrote in 2009 (Res Microbiol) - "The...question is: Why do all Rex regulated genes not behave in the same way? Obviously, there is fine adjustment in expression of many anaerobically induced genes that need, in addition to inactivation of Rex, a second regulatory protein that activates their transcription under anaerobic conditions."

This is an excellent point. Indeed, the Rex-regulated genes are diverse amongst Gram-positive organisms (Ravcheev 2011, JBac), likely corresponding to the diverse niches and metabolic capabilities of different organisms. As stated above, expression of many Rex-regulated genes are most likely under control of one or more secondary regulatory proteins. For example, it has been demonstrated in *L. monocytogenes* that transcription of *bsh* and *inlAB* is activated by PrfA and SigB in anaerobic conditions, when Rex-mediated repression is alleviated (Begley 2005, Dussurget 2002, Kazmierczak 2003). We look forward to investigating these overlapping regulatory circuits in future studies.

Minor comments:

1. misspelling in Fig S1 line 161
2. Correct fonts in figure legend Fig 2S

Thank you for identifying these errors.
