# Supplementary material for: The redox-responsive transcriptional regulator Rex represses fermentative metabolism and is required for Listeria monocytogenes pathogenesis
Source: PLoS Pathog. 2021 Aug 16;17(8):e1009379. doi: 10.1371/journal.ppat.1009379 (PMC8389512; doi:10.1371/journal.ppat.1009379)
Supplement: Attachment — Submitted filename: Response to resubmission reviews FINAL.docx [file ppat.1009379.s008.docx]

**Part II – Major Issues: Key Experiments Required for Acceptance**
Please use this section to detail the key new experiments or modifications of existing experiments that should be absolutely required to validate study conclusions.

Reviewer #1: (No Response)

Reviewer #2: N/A

Reviewer #4: 1. Aerobic/fermentative metabolism by the rex mutant
- The aerobic growth defect of the rex mutant, although statistically significant, is very small. BHI likely has excessive fermentable substrates, allowing both fermentation and oxidative phosphorylation to occur. I suggest that the authors revisit aerobic growth in a defined medium, as they also briefly mentioned in the discussion. This is an easy experiment that will substantially strengthen the conclusion.

Preliminary growth experiments in a common chemically defined medium show that the ∆*rex* mutant exhibits a similar small defect as when grown in BHI (results below). Therefore, we have removed line 298 (in marked-up version of the manuscript) from the discussion. While we initially predicted the ∆*rex* mutant would have a larger defect in this medium, more research brought this assumption into question. In fact, the metabolism of *L. monocytogenes* has never been characterized in this chemically defined medium, which contains almost 5 times the concentration of glucose as BHI (55mM versus 12mM). Additional experimentation is required to ascertain the metabolic pathways necessary for growth in defined medium before we could potentially modify the recipe to observe a large ∆*rex* growth defect. However, we feel those experiments would not impact our overall conclusion that Rex functions to repress fermentative metabolism.

- In Fig. 1G: ATP production by WT and delta-rex in the absence of oxygen should be presented as relative to WT grown aerobically. If increased fermentation reduces ATP synthesis, then anaerobic WT should have less ATP than aerobic WT.

We appreciate this recommendation, which has improved the presentation of these data Fig. 1G was changed as the reviewer suggested and Lines 161-163 (in marked-up version of the manuscript) were modified to reflect the data presented.

2. Bile survival phenotype
- The bile exposure experiments were performed for 24 hours. This seems like a very long time, in comparison to 8 hours of exposure described in Dowd et al. 2011. I am concerned that such a long exposure may produce artifactual data due to spontaneous suppressors and altered metabolism in prolonged stationary phase.

While the Dowd *et al.* 2011 paper performed experiments for up to 8 hours, several other papers have investigated the bile response of *L. monocytogenes,* analyzing time points from 15 minutes to 72 hours (Sleator *et al.* 2005; Begley *et al.* 2002; Begley *et al.* 2005; Dussurget *et al.* 2002; Quillin *et al.* 2011; Begley *et al.* 2009). In our experiments, the strains exposed to neutral bile do not grow or die so spontaneous suppressors are unlikely. In acidic bile, we start to see killing as early as 3 hours, and the difference in survival between strains increases over time. Thus, we present data at 24 hours, in which the difference between strains is greatest.

Additionally, data in Figure 2 do not prove that increased bile resistance in the delta-rex mutant is due to increased bsh expression, since this was not directly quantified under the tested condition. I have read the authors’ explanation that they could not obtain quality RNA from bile-treated cultures, and that bsh over-expression was technically difficult. As an alternative approach to evaluate the bsh regulation by Rex, why not test the delta-rex delta-bsh pPL2-bsh strain (driven by a chemically-inducible promoter), which is not subject to Rex regulation? If performed, the experiment will strengthen the conclusion and show a physiological relevance for bsh regulation by Rex.

We modified the text to more accurately describe the findings from our experiments (lines 190-191 in marked-up version of the manuscript). While we appreciate the idea to utilize a chemically-inducible promoter to increase *bsh* expression, we do not feel this would contribute to our overall conclusions for two reasons. First, the role of *L. monocytogenes* Bsh has been extensively studied and it has been well-established that its expression provides protection against acidified bile (Dowd *et al.* 2011; Begley *et al.* 2005; Dussurget *et al.* 2002). Second, and most importantly, the resistance to bile we observed *in vitro* is not relevant to the dramatic defect observed in the gallbladder of infected animals, which is the novel finding of our work.

3. Tissue culture infection
- In Fig. 3, what are the Caco-2 and Huh7 infection rates by WT? I recall from the literature that 10403S invades Caco-2 cells at ~1%. If so, the small increase in invasion by the delta-rex mutant may not have much biological significance.

Yes, in the literature and in our experiments, the infection rate of wt is typically between 1% and 3% in both Caco-2 and Huh7 cells. As we state in lines 315-319 we agree with you that this increase is unlikely to have an impact during oral infection of a mouse.

- Similar to the bile survival data, Figure 3 does not prove that increased Caco-2 and Huh7 invasion by delta-rex is due to increased inlAB expression, which was not quantified for Listeria cultures grown for infection – a different condition than that for RNASeq and qPCR experiments. Furthermore, at 2 hours post infection, Listeria would have gotten out of the phagosome, so the data here reflect both endocytosis and vacuolar survival/escape. Finally, growth curves in Figure 4 and mouse infection data in Figure 5 suggest that the small difference exhibited by delta-rex in early time points does not impact infection kinetics or outcomes. I’m not asking the authors to do more experiments here, but I’d suggest that they refrain from attributing the small phenotype in Figure 3 to inlAB expression and discuss the physiological relevance of this data.

If the increase in invasion by ∆*rex* was due to factors other than *inlAB* expression, we would expect the ∆*rex∆inlAB* mutant to have an intermediate phenotype compared to wt and ∆*inlAB.* We agree that at 2 hours post-infection some bacteria will be within or escaped from the vacuole, however the methods we used to determine invasion are common in the *Listeria* literature (Kuhbacher *et al.* 2015, *Methods In Molecular Biology*). As we state in the discussion, while we don’t believe the small invasion increase by ∆*rex* contributes during oral infection of a mouse, we do hypothesize that Rex, along with other transcriptional regulators such as SigB and PrfA, converge for appropriate and efficient regulation of *inlAB.*

- Because the increase in Caco2 and Huh7 initial infection did not translate to GI tract infection, the discussion in lines 344-347 and 353-356 seems moot and misleading. I suggest this to be removed.

We respectfully disagree. These sentences accurately describe our conclusions from the *in vitro* experiments, which is clearly stated. We then go on to point out the discrepancies between our *in vitro* findings and the *in vivo* results, which is important not only for our work, but for the field in general. However, we modified lines 320 and lines 328-330 to further highlight the key differences between *in vitro* and *in vivo* results.

4. Mouse infection studies
- Data in figure 5 are the most substantial and novel aspect of the manuscript. I think that the lack of delta-rex phenotype in GI tract infection is consistent with the microaerophilic/anaerobic condition in the GI tract, under which Rex does not repress target genes.

We agree with the reviewer’s comment. As we stated in lines 332-334, we hypothesize that the lack of phenotype in the GI tract in mice infected with ∆*rex* is due to Rex repression being alleviated in wt bacteria in this environment.

- I find data in Fig 5F-G-H and Fig. S4 very intriguing, as they suggest that the delta-rex mutant is defective for replication in systemic organs, even though it initially reaches these organs equally well compared to WT. I noticed that the authors have performed a preliminary intravenous infection for WT and delta-rex, and found them to exhibit similar burdens in the spleen and liver at 48hpi. This data is consistent with Fig. S4 in which delta-rex defect only occurs between 2 and 4 dpi. So I strongly encourage the authors to repeat intravenous infection for more replicates and assess burdens at 2 and 4dpi. Validating the importance of Rex during systemic infection would substantially increase the impact of the manuscript.

We agree that these results are intriguing. Ongoing work is aimed at elucidating the role of Rex in dissemination, replication in systemic organs, and potential interaction with immune components. However, these experiments are outside the scope of the current manuscript.

**Part III – Minor Issues: Editorial and Data Presentation Modifications**

Reviewer #1: In the discussion, I would suggest rephrasing the following sentence in the final version:
P17L342-344 “While InlAB-mediated invasion is required for invasion of non-phagocytes in cell culture, it has been reported that neither of these adhesins are required for successful infection within a mouse” might be amended to “While InlAB-mediated invasion is required for invasion of non-phagocytes in cell culture, it has been reported that neither of these adhesins are required for successful intestinal infection in mice.” (or "oral infection in the mouse listeriosis model").
Indeed, while mouse E-cadherin is insensitive to InlA, mouse Met is a target of InlB. Only the absence on the InlB-mediated pathway in the gut makes mice insensitive to both InlA and B during oral infection. In contrast, the intravenous route is InlB-sensitive in mice.

We appreciate this clarification suggested by the reviewer and have changed lines 318-320 as the reviewer proposed. (Now lines 317-319 in marked-up version of the manuscript).

Reviewer #2: N/A

Reviewer #4: None
